# Mode‐of‐Action and Human Relevance Assessment for Diisononyl Phthalate‐Induced Liver Tumors in Rodents

**DOI:** 10.1002/jat.70223

**Published:** 2026-05-05

**Authors:** Amanda N. Buerger, Melissa M. Heintz, Laurie C. Haws, Chad M. Thompson

**Affiliations:** ^1^ ToxStrategies LLC Cincinnati Ohio USA; ^2^ ToxStrategies LLC Asheville North Carolina USA; ^3^ ToxStrategies LLC Austin Texas USA; ^4^ ToxStrategies LLC Houston Texas USA

**Keywords:** diisononyl phthalate (DINP), liver, mode of action (MOA), peroxisome proliferator‐activated receptor alpha (PPARα)

## Abstract

Diisononyl phthalate (DINP) is a high molecular weight phthalate and high production volume chemical. DINP's carcinogenic potential has been investigated in four rodent bioassays, with liver tumors observed in three of the studies. Authoritative assessments have hypothesized that DINP acts through the peroxisome proliferator‐activated receptor alpha (PPARα) activator‐induced mode of action (MOA) for rodent hepatocarcinogenesis. However, these assessments reported disparate conclusions regarding the human relevance of this rodent‐specific MOA, and alternative MOAs for DINP‐induced rodent liver tumors have been proposed, albeit with limited evidence. Herein, the MOA for DINP‐induced rodent liver tumors is assessed according to the MOA framework for PPARα activator‐induced rodent hepatocarcinogenicity, which includes four key events (KEs). Findings demonstrate strong evidence that DINP induces KE 1 (PPARα activation) and KE 3 (perturbation of cell growth and survival) in vitro and in vivo in wild‐type and PPARα‐null rodent models. KE 2 and KE 4 are reasonably inferred, given the strong data for KE 1, KE 3, and the adverse outcome (liver tumors). Alternative MOAs, including genotoxicity, endocrine disruption, other nuclear receptor activation, and cytotoxicity, are not supported by the evidence. The human and nonhuman primate evidence for DINP supports the lack of human relevance of this MOA, as well as the divergent PPARα‐mediated pathways between species, with evidence in humans limited to PPARα activation (KE 1). Overall, the weight of evidence strongly supports that DINP acts through the nonhuman‐relevant PPARα MOA for rodent hepatocarcinogenesis; therefore, rodent liver tumors and upstream KEs 2–4 should not serve as the basis for human health risk conclusions.

## Abstract 2

The weight of evidence strongly supports diisononyl phthalate (DINP) acts through the peroxisome proliferator‐activated receptor alpha (PPARα) mode of action (MOA) for rodent hepatocarcinogenesis. Evidence does not support DINP acts through alternative MOAs (e.g., genotoxicity and cytotoxicity), and DINP‐specific data support the lack of human relevance of rodent liver tumors and upstream key events (KEs) 2–4 of this MOA. Therefore, DINP‐mediated liver effects in rodents following PPARα activation (KE 1) should not serve as the basis for human health risk conclusions.

## 1 Introduction

Diisononyl phthalate (DINP) is a common name for the high molecular weight and complex mixture of diester phthalates, which is available as two types: 1,2‐benzenedicarboxylic acid, esterified with either branched alcohols (with alkyl side chains of C8–C10, C9 rich) or isononyl alcohols. DINP is used primarily as a plasticizer in the manufacture of polyvinyl chloride (PVC) and in a variety of commercial and consumer products, including plastic and rubber products, adhesives and sealants, paints and coatings, electrical and electronic products, automotive and fuel products, and building and construction materials (US EPA 2025e). DINP belongs to the larger class of synthetic phthalate esters that, in addition to occupational settings, can be found in the environment and in human urine samples (US EPA 2025e).

Authoritative and regulatory assessments of phthalates have focused largely on assessing reproductive and/or carcinogenic potential. As a chemical class, ortho‐phthalates have been studied widely as potential male reproductive toxicants, often through their potential to disrupt endocrine pathways. Recently, however, it was concluded that DINP does not meet the European Chemical Agency (ECHA)/European Food Safety Authority (EFSA) criteria to be classified as an endocrine disrupter (Lea et al. 2025), and ECHA's Committee for Risk Assessment determined no reproductive or developmental toxicity classification was warranted for DINP (ECHA 2018). Authoritative and regulatory assessments of DINP conducted to date have included assessment of carcinogenic potential, and DINP is set to undergo evaluation by the International Agency for Research on Cancer (IARC) in 2026 (EC 2003; HC 2015; OEHHA 2013; US EPA 2025a). The carcinogenicity of DINP has been investigated in four chronic rodent bioassays (Biodynamics 1986, 1987; Covance 1998a, 1998b; Lington et al. 1997). Increased incidences of liver adenomas and/or carcinomas were observed in three studies, in B6C3F1 mice, F344 rats, and Sprague–Dawley (SD) rats (Biodynamics 1987; Covance 1998a, 1998b). The lack of increased liver adenomas and/or carcinomas observed in a second study in F344 rats was likely due to comparatively lower doses than the positive studies (Lington et al. 1997). Data indicate that DINP (and its metabolites) are not direct‐acting genotoxicants, and thus, the rodent liver tumors are the result of nongenotoxic mechanisms (EC 2003; ECHA 2013; Lea et al. In Press; OEHHA 2013; US EPA 2025a). The United States Environmental Protection Agency (US EPA) recently conducted a risk evaluation of DINP and concluded that the liver tumors were the result of a peroxisome proliferator‐activated receptor alpha (PPARα) mode of action (MOA) (US EPA 2025a). Other organizations have reached similar conclusions (ECHA 2013; HC 2015; NICNAS 2012; US CPSC 2001).

It is widely accepted that nongenotoxic chemicals that act through PPARα receptor activation lack human relevance (Corton et al. 2014, 2018; Felter et al. 2018; Yamada et al. 2025). However, US EPA (2025a, 2025e) concluded that DINP is “Not Likely to be Carcinogenic to Humans at doses below levels that do not result in PPAR activation” (p. 38). This conclusion is at odds with data that support that PPARα activation in humans results in qualitatively different downstream signaling processes from rodents, such that human PPARα activation does not result in signaling pathways related to cell cycle that play an integral role in liver tumorigenesis in rodents. The present article reports an MOA analysis of DINP‐induced liver tumors in rodents and assesses the human relevance of these tumors by applying principles from established frameworks (Boobis et al. 2006; Meek et al. 2014; US EPA 2005). The authors expect that these analyses will strengthen the mechanistic and human relevance foundation for future evaluations of DINP carcinogenicity.

## 2 Carcinogenesis of DINP

The assessment of DINP carcinogenicity in animals has been the subject of four 2‐year dietary studies, including two studies in male and female F344 rats (Biodynamics 1986; Covance 1998b; Lington et al. 1997): one study in male and female SD rats (Biodynamics 1987) and one study in male and female B6C3F1 mice (Covance 1998a). US EPA recently concluded that DINP is not likely to be carcinogenic to humans at doses below those that do not activate PPARα (US EPA 2025a). This conclusion was based, in part, on evidence for PPARα‐mediated liver tumors in rodents and the lack of evidence for an association between DINP and cancer in humans. A thorough review of the evidence for PPARα‐mediated liver tumors in rodents is presented herein, and a systematic evaluation of the association between DINP and cancer in humans is presented by Lea et al. (In Press).

In addition to the rodent liver tumors, kidney tumors were observed in male F344 rats, and mononuclear cell leukemia (MNCL) was observed in aging F344 rats (Biodynamics 1986; Covance 1998b; Lington et al. 1997), but not SD rats or mice (Biodynamics 1987; Covance 1998a). The MOA and human relevance of these extrahepatic tumors are beyond the scope of this article but appear to be unrelated to PPARα activity as discussed elsewhere (Lea et al. In Press). Liver tumors were observed in three of the four 2‐year oral DINP rodent cancer bioassays. The liver tumor incidences in mice and rats are shown in Tables 1 and 2, respectively. In mice, liver tumor incidence was elevated only at ≥ 742 and ≥ 336 mg/kg/day in males and females, respectively. In rats, liver tumor incidence was not increased in Lington et al. (1997) (also Biodynamics 1986), where the mg/kg/day doses in male and female F344 rats did not exceed 307 and 375 mg/kg/day, respectively. In Covance (1998b), liver tumor incidence was increased significantly in male and female F344 rats at 733 and 885 mg/kg/day, respectively. In SD rats, liver tumor incidence was increased significantly only in female rats at ≥ 331 mg/kg/day. The evidence related to the MOA underlying the liver tumors in rodents, and to the human relevance of that MOA, is described in the following sections.

**TABLE 1:** Liver tumor incidence in mice administered DINP in the diet for up to 104 weeks.

	Males					Females				
DINP in diet (mg/kg/day)	0 ppm (0)	500 ppm (90)	1500 ppm (276)	4000 ppm (742)	8000 ppm (1560)	0 ppm (0)	500 ppm (112)	1500 ppm (336)	4000 ppm (910)	8000 ppm (1888)
Adenoma	10/70	7/67	8/66	15/65	13/70	2/70	4/68	5/68	4/67	18/70^a^
Carcinoma	10/70	8/67	10/66	17/65	20/70^a^	1/70	2/68	5/68	7/67^a^	19/70^a^
Combined	16/70	13/67	18/66	28/65^a^	31/70^a^	3/70	5/68	10/68^a^	11/67^a^	33/70^a^

^a^ Statistically significant difference from controls (*p* ≤ 0.05) by logistic regression analysis.

*Source:* Covance 1998b (incidence as presented by US CPSC (2001) for mice that were examined for liver histopathology upon unscheduled deaths, interim sacrifice at 79 weeks, and terminal sacrifice).

**TABLE 2:** Liver tumor incidence in rats administered DINP in the diet for up to ~104 weeks.

	Males							Females						
%DINP in diet	0	0.03	0.05	0.15	0.30	0.60	1.2	0	0.03	0.05	0.15	0.30	0.60	1.2
ppm DINP in diet (mg/kg/day)	0 (0)	300 (15)	500 (29)	1500 (88)	3000 (152)	6000 (307) (359)	12,000 (733)	0 (0)	300 (18)	500 (36)	1500 (109)	3000 (184)	6000 (375) (442)	12,000 (885)
Biodynamics (1986)/Lington et al. (1997) (F)^a^	3/81	1/80	—	—	1/80	4/80	—	1/81	2/81	—	—	0/80	2/80	—
Covance (1998a)^b^ (F)^a^	5/65	—	3/50	2/50	—	7/65	18/65^c^	1/65	—	1/49	0/50	—	2/65	8/65^c^

ppm DINP in diet (mg/kg/day)	0 (0)	—	500 (27)	—	5000 (271)		10,000 (553)	0 (0)	—	500 (33)	—	5000 (331)		10,000 (672)
Biodynamics (1987) (SD)^d^	2/70	—	2/69	—	6/69		4/70	0/70	—	0/70	—	5/70^c^		7/70^c^

Abbreviations: F, F344; SD, Sprague–Dawley.

^a^ Combined tumor incidence.

^b^ Incidence presented for mice that were examined for liver histopathology upon unscheduled deaths, interim sacrifice at 79 weeks, and terminal sacrifice as presented by US CPSC (2001).

^c^ Statistically significant difference from controls.

^d^ Carcinoma incidence.

## 3 MOA Assessment

The evidence that DINP acts through the PPARα MOA for liver tumors in rodents is evaluated according to the PPARα MOA framework described by Corton et al. (2014, 2018). This MOA assessment was conducted according to cancer assessment guidance from the US EPA and International Programme on Chemical Safety (IPCS) and included consideration of animal and human data that map to key events (KEs) and the adverse outcome (AO), consideration of alternative MOAs, and incorporation of aspects of the Modified Bradford Hill criteria, where possible (Boobis et al. 2006; Meek et al. 2014; US EPA 2005). Data relevant to each of the four KEs of the MOA are presented, including any relevant evidence from rodents, nonhuman primates, and humans.

### 3.1 KE 1: PPARα Activation

PPARα activation is the molecular initiating event (MIE), or the first KE (KE 1), in the PPARα MOA for rodent liver tumors. Evidence relevant to KE 1 includes direct findings of PPARα binding and/or activation, as well as associative events indicative of PPARα activation, such as increased peroxisomal β‐oxidation (PBOX) and associated enzyme activity (e.g., palmitoyl‐CoA [PCO]) in the liver, histological evidence of peroxisome proliferation, and decreased circulating triglycerides (Corton et al. 2014, 2018). Notably, these associative events are not causal components of the MOA itself, but they are reliable markers of PPARα activation, with increased PBOX and peroxisome proliferation being rodent‐specific markers of PPARα activation that do not occur in humans following PPARα activation, due to differences in the transcriptional networks regulated by PPARα between rodents and humans.

Evidence of PPARα activation by DINP has also been demonstrated for its metabolite, monoisononyl phthalate (MINP), in two studies that used reporter assays (Table 3; additional study details found in Table S1) (Bility et al. 2004; Laurenzana et al. 2016). In a luciferase reporter assay, MINP activated mPPARα and hPPARα in mouse 3T3‐L1 cells, with increased sensitivity of the mPPARα (lowest activation concentration: 3 μM; maximal fold induction: 27.1 ± 1.9) compared to hPPARα (lowest activation concentration: 10 μM; maximal fold induction: 5.8 ± 2.1) (Bility et al. 2004). In an assay of hPPARα transfected into COS‐1 cells exposed to 0.1‐ to 100‐μM MINP, reporter activity was minimally increased by approximately 1.75‐fold above control at 100 μM (Laurenzana et al. 2016). DINP hPPARα activation in one ToxCast assay was negative (US EPA 2025a); however, no ToxCast data were available for MINP.

**TABLE 3:** Summary of toxicological evidence supporting KE 1 (PPARα activation and associative events) in the PPARα MOA for rodent liver tumors.^a^

	Rodents			Nonhuman primates and humans		
Effect	WoE result	Model	Reference(s)	WoE result	Model	Reference(s)
KE 1: PPARα activation						
Direct evidence						
PPARα activation	+	Mouse PPARα reporter assay; MINP	Bility et al. (2004)	+^b^	Human PPARα reporter assay; MINP	Bility et al. (2004); Laurenzana et al. (2016)
	+	Primary rat hepatocyte gene expression; DINP	De Abrew et al. (2015)	−	Human PPARα reporter assay; DINP	Laurenzana et al. (2016); US EPA ToxCast
*Associative Events*						
PBOX and ACO/PCO Activity	+	Mouse, diet, 4 days	Hazleton (1992)	−	Monkey, gavage, 2 weeks	Pugh et al. (2000)
	+	Rat, diet, 1 week	Covance (1998b)	−	Monkey, gavage, 13 weeks	Hall et al. (1999)
	+	Mouse, gavage or diet, 1 week	Valles et al. (2003)	−	Primary monkey hepatocytes; MINP	Kamendulis et al. (2002); Benford et al. (1986)
	−	PPARα‐null mice, gavage or diet, 1 week	Valles et al. (2003)	−	Primary monkey hepatocytes; DINP	Benford et al. (1986)
	+	Rat, diet, 2 weeks	Covance (1998b); Smith et al. (2000)	−	Primary human hepatocytes; MINP	Shaw et al. (2002)
	+	Mouse, diet, 2 weeks	Valles et al. (2003); Smith et al. (2000)	−	Primary human hepatocytes; DINP	Hasmall et al. (1999)
	+	Mouse, diet, 3 weeks	Valles et al. (2003)	−	Immortalized human cells (HLEC‐4); MINP	Kamendulis et al. (2002)
	−	PPARα‐null mice, diet, 3 weeks	Valles et al. (2003)			

+	Rat, diet, 3 weeks	BIBRA (1986)			
	+	Mouse, diet, 4 weeks	Kaufmann et al. (2002); Smith et al. (2000)			
	+	Mouse, diet, 31 days	Hazleton (1992)			
	+	Rat, diet, 4 weeks	Smith et al. (2000)			
	+	Mouse, diet, 90 days	Hazleton (1992)			
	+	Rat, diet, 13 weeks	Covance (1998b)			
	+	Mouse, diet 78 weeks	Covance (1998a)			
	+	Rat, diet, 78 weeks	Covance (1998b)			
	+	Mouse, diet, 104 weeks	Covance (1998a)			
	+	Rat, diet, 104 weeks	Covance (1998b)			
	+^c^	Primary rat hepatocytes; DINP	Hasmall et al. (1999); Benford et al. (1986)			
	+	Primary rat hepatocytes; MINP	Benford et al. (1986); Shaw et al. (2002); Kamendulis et al. (2002)			
	+	Primary mouse hepatocytes	Kamendulis et al. (2002)			
Peroxisome Proliferation	+	Rat, diet, 3 weeks	BIBRA (1986)			
	+	Mouse, diet, 4 weeks	Kaufmann et al. (2002)			
	+	Rat, diet, 14 weeks	Biodynamics (1982b)			
	−	Rat, diet, 104 weeks	Biodynamics (1986)/Lington et al. (1997)			
Hepatocellular hypertrophy and eosinophilic cytoplasm	−	Mouse, diet, 4 days	Hazleton (1992)	−	Monkey, gavage, 2 weeks	Pugh et al. (2000)
	−	Rat, diet, 1 week	Covance (1998b)	−	Monkey, gavage, 13 weeks	Hall et al. (1999)
	+	Rat, diet, 2 weeks	Covance (1998b)			
	+	Rat, diet, 3 weeks	BIBRA (1986)			
	+	Mouse, diet, 4 weeks	Hazleton (1991a)			
	+	Mouse, diet, 31 days	Hazleton (1992)			
	+	Mouse, diet, 90 days	Hazleton (1992)			
	+	Rat, diet, 13 weeks	Biodynamics (1982b, 1982c); Hazleton (1971, 1991b); Covance (1998b)			
	−	Rat, diet, 6 months	Biodynamics (1986)/Lington et al. (1997)			
	−	Rat, diet, 12 months	Biodynamics (1986)/Lington et al. (1997)			
	+	Rat, diet, 78 weeks/18 months	Covance (1998b); Biodynamics (1986)/Lington et al. (1997)			
	+	Mouse, diet, 79 weeks	Covance (1998a)			
	+	Rat, diet, 104 weeks	Covance (1998b); Biodynamics (1986)/Lington et al. (1997)			
	+	Mouse, diet, 104 weeks	Covance (1998a)			
	+	Rat, diet, one‐generation reprotoxicity study	Waterman et al. (2000)			
	+	Rat, diet, two‐generation reprotoxicity study	Waterman et al. (2000)			
Increased liver weight	+	Rat, diet, 1 week	Covance (1998b); Biodynamics (1982a)	−	Monkey, gavage, 2 weeks	Pugh et al. (2000)
	+/−	Mouse, gavage or diet, 1 week	Valles et al. (2003); Kaufmann et al. (2002)	−	Monkey, gavage, 13 weeks	Hall et al. (1999)/Hu
	−	PPARα‐null mice, gavage or diet, 1 week	Valles et al. (2003)			
	+/−	Rat, gavage or diet, 2 weeks	Smith et al. (2000); Covance (1998b); Kwack et al. (2010)			
	+	Mouse, diet, 2 weeks	Valles et al. (2003); Smith et al. (2000)			
	+	Rat, diet, 3 weeks	BIBRA (1986)			
	+	Mouse, diet, 3 weeks	Valles et al. (2003)			
	+/−	PPARα‐null mice, diet, 3 weeks	Valles et al. (2003)			
	+	Rat, oral or diet, 4 weeks	Smith et al. (2000); Kwack et al. (2009); Midwest Research Institute (1981)			
	+	Mouse, diet, 4 weeks	Kaufmann et al. (2002); Smith et al. (2000); Hazleton (1991a, 1991b)			
	+	Mouse, dermal, 4 weeks	Liang and Yan (2020)			
	+	Mouse, diet, 90 days	Hazleton (1992)			
	+	Rat, diet, 13 weeks	Biodynamics (1982b, 1982c); Hazleton (1971, 1991b); Covance (1998b)			
	+	Rat, diet, 6 months	Biodynamics (1986)/Lington et al. (1997)			
	+	Rat, diet, 12 months	Biodynamics (1986)/Lington et al. (1997); Biodynamics (1987)			
	+	Rat, diet, 18 months/78 weeks	Biodynamics (1986)/Lington et al. (1997); Covance (1998b)			
	+	Mouse, diet, 78 weeks	Covance (1998a)			
	+	Rat, diet, 104 weeks	Biodynamics (1986)/Lington et al. (1997); Covance (1998b); Biodynamics (1987)			
	+	Mouse, diet, 104 weeks	Covance (1998a)			
	+	Rat, diet, one‐generation reprotoxicity study	Waterman et al. (2000)			
	+	Rat, diet, two‐generation reprotoxicity study	Waterman et al. (2000)			
Decreased circulating triglycerides	+	Rat, diet, 1 weeks	Biodynamics (1982a)	−	Monkey, gavage, 2 weeks	Pugh et al. (2000)
	−	Rat, gavage, 2 weeks	Kwack et al. (2010)			
	+/−	Rat, diet, 3 weeks	BIBRA (1986)			
	+/−	Rat, gavage, oral, or diet, 4 weeks	Kwack et al. (2009); Midwest Research Institute (1981); Ore et al. (2022)			
	+	Rat, diet, 13 weeks	Biodynamics (1982b), (1982c)			
	+/−	Rat, diet, 6 months	Biodynamics (1987); Biodynamics (1986)/Lington et al. (1997)			
	+/−	Rat, diet, 12 months	Biodynamics (1987); Biodynamics (1986)/Lington et al. (1997)			
	−	Rat, diet, 18 months	Biodynamics (1987); Biodynamics (1986)/Lington et al. (1997)			
	−	Rat, diet, 24 months	Biodynamics (1987); Biodynamics (1986)/Lington et al. (1997)			

^a^ A more detailed summary of these studies is provided in Table S1.

^b^ hPPARα had a positive but substantially lower sensitivity relative to mPPARα in Bility et al. (2004).

^c^ Negative result reported by study authors to be related to lack of metabolism in vitro.

Transcriptomic data further support that DINP is a PPARα activator. Microarray expression data from primary rat hepatocytes treated with DINP or prototypical PPARα agonists WY14643 and clofibrate or prototypical constitutive androstane receptor (CAR)‐agonist phenobarbital for 24 or 48 h demonstrated that the transcriptomic profile of DINP was consistent with that of the prototypical PPARα agonists WY14643 and clofibrate (De Abrew et al. 2015). Evaluation of these transcriptomic data with biomarker gene sets for PPARα activation (Podtelezhnikov et al. 2020), cytotoxicity (Corton et al. 2020), and cell proliferation (Corton et al. 2024) clearly demonstrated that DINP, WY14643, and clofibrate activate the PPARα gene set, unlike phenobarbital, the prototypical CAR‐agonist (Figure 1).

**FIGURE 1:**
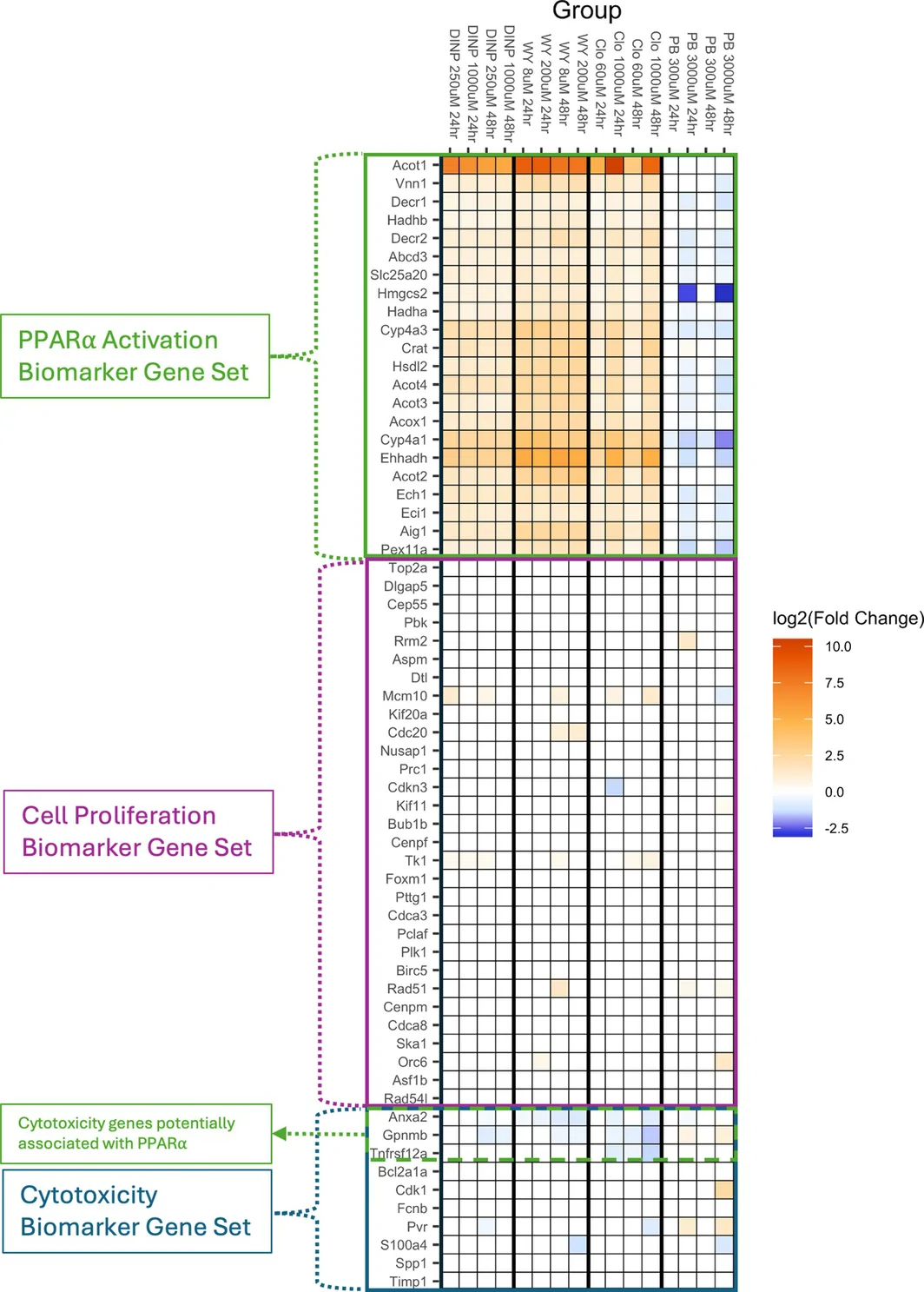
Heatmap of gene expression data from De Abrew et al. (2015) for PPAR α activation, cell proliferation, and cytotoxicity biomarkers in primary rat hepatocytes exposed to DINP, WY14643 (WY), clofibrate (Clo), or phenobarbital (PB) for 24 or 48 h. Significant (FDR < 5%, no log2[fold change]threshold) differentially expressed genes (rows) are indicated by orange (upregulated) or blue (downregulated) colors; intesity of colors is based on the fold change value for the gene within a treatment group (column). White cells indicate that a gene was not significantly altered for a chemical treatment group following exposure compared to respective controls. The PPAR α activation biomakers gene set was developed using data from rats (Podtelezhnikov et al. 2020), the cytotoxicity biomakers gene set was developed using data from rats (Corton et al. 2020) and the cell proliferation biomaker gene set was developed using data from mice, rats, and human cells (Corton et al. 2024).

The evidence of PPARα activation is supported further by a large body of in vivo and in vitro associative events: increased liver PBOX and associated enzyme activity (e.g., PCO) in rodents, histological evidence of peroxisome proliferation in rodents, and decreased circulating triglycerides in rodents and humans. Regarding PBOX and associated enzyme activity in the liver, PBOX and increased acetyl‐CoA (ACO) and PCO activity was observed across 10 in vivo studies in rats and wild‐type (wt) mice exposed to DINP from 4 days to 104 weeks, whereas hepatic PBOX and PCO activity was not observed in PPARα‐null mice (Table 3; additional study details found in Table S1) (BIBRA 1986; Covance 1998a, 1998b; Hazleton 1992 as cited by US EPA 2025; Kaufmann et al. 2002; Smith et al. 2000; Valles et al. 2003). The essentiality of PPARα in PBOX and associated enzyme activity was demonstrated through a series of studies using wt (B6C3F1 and SV129) and PPARα‐null mice administered DINP via diet (0 or 8000 ppm) for 1 to 3 weeks or by gavage (1000 mg/kg/day) for 1 week (Valles et al. 2003). These studies demonstrated increased ACO and PCO activity and expression of genes and proteins related to PBOX in wt but not PPARα‐null mice (Valles et al. 2003). Conversely, two studies using nonhuman primates found no PBOX or PCO activity after 2 weeks of daily DINP gavage at 500 mg/kg/day or 13 weeks up to 2500 mg/kg/day, respectively (Hall et al. 1999; Pugh et al. 2000). Similar species differences in this PPARα‐related associative event are observed in vitro, where administration of DINP or MINP consistently increased PBOX and PCO activity in primary rat and mouse hepatocytes but not in primary nonhuman primate or human hepatocytes or immortalized human cell lines (Benford et al. 1986; Hasmall et al. 1999; Kamendulis et al. 2002; Shaw et al. 2002). These species‐specific differences in indicators of PPARα activation are consistent with the divergent gene sets regulated by PPARα in rodents versus humans and nonhuman primates.

Histopathological evidence of DINP‐induced PPARα activation includes direct assessment of peroxisome proliferation and hepatocellular hypertrophy. Peroxisomal proliferation was examined through electron or light microscopy by counting peroxisomes or measuring the area of peroxisomes in three rodent studies for DINP. Peroxisomal proliferation has been observed at 3 weeks (≥ 2195 mg/kg/day) and 13 weeks (≥ 460 mg/kg/day) in rats and at 4 weeks (≥ 300 mg/kg/day) in mice (Table 3; additional study details found in Table S1) (BIBRA 1986; Biodynamics 1982b; Kaufmann et al. 2002). Electron microscopic evaluation of F344 rats exposed to ~400 mg/kg/day DINP for 104 weeks found no evidence of peroxisomal proliferation (Biodynamics 1986; Lington et al. 1997). However, the absence of peroxisomal proliferation is consistent with the absence of a statistically significant increase in liver tumors in the study. Thus, the weight of evidence (WoE) from these studies supports that DINP elicits peroxisome proliferation in rodents at high doses during shorter exposures and at lower doses during longer exposures in mice. Absence of peroxisome proliferation at 2 years in the Lington et al. (1997) study is consistent with the lack of liver tumors observed in that study and bears hallmarks of a weak PPARα activator.

Hepatocyte hypertrophy, also referred to as hepatocellular enlargement, occurs as the result of enzymatic induction from PPARα activation in rodents and is often characterized by eosinophilic and granular cytoplasm (NTP 2019; NTP and Maronpot 2013; Thoolen et al. 2010). Evidence supporting this associative event was observed in all studies in which hepatocellular hypertrophy was evaluated (Table 3; additional study details found in Table S1) (BIBRA 1986; Biodynamics 1982a, 1982b, 1982c, 1986; Covance 1998a, 1998b; Hazleton 1971, 1991a, 1991b; Hazleton 1992 as cited by US EPA 2025; Lington et al. 1997; Waterman et al. 2000). Hepatocellular enlargement and/or eosinophilic changes were observed consistently after ≥ 2 weeks of DINP exposure. Only in one chronic bioassay in F344 rats were no liver histopathological indications of these changes observed after ≥ 2 weeks (i.e., 6 and 12 months); however, by 18 and 24 months, there was “subtle” hepatocellular enlargement at the highest dose (0.6%; 307/375 mg/kg/day) (Biodynamics 1986; Lington et al. 1997). This subtle change at the highest dose only after chronic DINP exposure is consistent with the lack of peroxisome proliferation and significant liver tumors observed in the Biodynamics (1986)/Lington et al. (1997) study. Importantly, no hepatocellular hypertrophy or cytoplasmic alterations were observed in nonhuman primates exposed to DINP for 2 weeks at 500 mg/kg/day or 13 weeks at up to 2500 mg/kg/day (Hall et al. 1999; Pugh et al. 2000).

Consistent with hepatocellular hypertrophy, increased liver weight is observed consistently in studies on PPARα activators, including DINP. Increased liver weight can result from both hepatocellular hypertrophy and hyperplasia, the latter of which is a component of KE 3 (perturbation of cell growth and survival). Notably, studies indicate hepatocellular hypertrophy and increased liver weight after short‐ and long‐term exposure to DINP, whereas cell proliferation (e.g., 5‐bromo‐2′‐deoxyuridine [BrdU] labeling) is observed only at early time points. Increased liver weight was observed consistently in rodent studies of DINP, including across nine studies in wt mice from seven publications/reports at ≥ 2 weeks DINP exposure, with mixed results at 1 week (Table 3; additional study details found in Table S1) (Covance 1998a; Hazleton 1991a, 1992; Hazleton 1992 as cited by the US EPA 2025; Kaufmann et al. 2002; Liang and Yan 2020; Smith et al. 2000; Valles et al. 2003) and in 15 of 16 studies from 15 publications/reports in rats following ≥ 1 week DINP exposure (Table 3; additional study details found in Table S1) (BIBRA 1986; Biodynamics 1982a, 1982b, 1982c, 1986, 1987; Covance 1998b; Hazleton 1971, 1991b; Kwack et al. 2009, 2010; Lington et al. 1997; Midwest Research Institute 1981; Smith et al. 2000; Waterman et al. 2000). A series of dietary studies in wt and PPARα‐null mice administered DINP at up to ~1200 mg/kg/day (8000 ppm) showed increased relative liver weights in wt male and female mice at 1, 2, and 3 weeks of exposure. In contrast, increased relative liver weight was observed in female PPARα‐null mice after 3 weeks of exposure (Valles et al. 2003); nevertheless, Valles et al. (2003) concluded that, overall, “PPARα mediates effects of DINP previously linked to liver cancer, including increases in hepatocyte proliferation and peroxisome proliferation.” As was observed with hepatocellular hypertrophy, there was no increase in liver weight in two studies of nonhuman primates exposed to DINP for 2 and 13 weeks (Hall et al. 1999; Pugh et al. 2000).

Decreased circulating triglycerides, though itself not likely a contributor to PPARα‐induced hepatocarcinogenesis, is an associative event of this MOA because it is an indicator of PPARα activation in rodents and humans. The rodent data for decreased circulating triglycerides following DINP exposure are equivocal (Table 3; additional study details found in Table S1) (BIBRA 1986; Biodynamics 1982a, 1982b, 1982c, 1987; Kwack et al. 2009, 2010; Midwest Research Institute 1981; Ore et al. 2022). No evidence of reduced circulating triglycerides was observed in a 2‐week study of nonhuman primates administered a daily gavage dose of DINP at 500 mg/kg/day (Pugh et al. 2000). Similarly, the majority of epidemiological studies that evaluated DINP exposure and potential associations with blood lipids did not find any associations with triglycerides or cholesterol, with only a few findings of decreased LDL and triglycerides with DINP exposure (Berman et al. 2022; Trasande and Attina 2015; Zhang et al. 2018). The lack of findings related to decreased circulating triglycerides in nonhuman primates and humans, and the equivocal results in rodents, are consistent with DINP as a weak PPARα activator.

Overall, the direct evidence for mPPARα activation by MINP in vitro, coupled with the strong in vivo evidence for the associative events of PBOX and ACO/PCO activity, peroxisome proliferation, hepatocellular hypertrophy and eosinophilic cytoplasm, and increased liver weight following DINP exposure, strongly supports a finding that DINP exposure in rodents activates PPARα, the first KE in the PPARα MOA for rodent liver tumors. The direct evidence of hPPARα activation by MINP, the in vitro assays in human and nonhuman primate hepatocytes exposed to DINP and MINP, and the in vivo evidence in nonhuman primates support a decreased sensitivity and/or responsiveness of human and nonhuman primate PPARα, as well as the divergent pathways controlled by rodent and human PPARα.

### 3.2 KE 2: Alteration of Cell Growth Pathways

PPARα activation is well‐documented to cause alterations in cell growth pathways in rodents (Corton et al. 2014, 2018). Given the evidence for KE 1 in rodent studies, alterations in cell signaling are expected to arise from downstream PPARα transcriptional networks. Furthermore, evidence for increased hepatocellular proliferation (see next section on KE 3) implies alterations in cell growth pathways. For example, rats exposed to 12,000 ppm DINP in diet exhibited significant increases in BrdU labeling index after 1 week of exposure (Covance 1998b). These changes were accompanied by increases in PCO activity and increased liver weight, both of which are associated with PPARα activation and peroxisomal proliferation. Increased liver weight can also be associated with chemicals that induce cytotoxicity and regenerative hyperplasia, such as carbon tetrachloride (Cohen et al. 2023); however, evidence of chronic liver injury such as overt cytotoxicity and fibrosis has not been observed following DINP exposure (see Section 5.5). Studies in wt and PPARα‐null mice exposed at concentrations up to 8000 ppm (~1200 mg/kg/day) DINP have reported significant increases in BrdU labelling index in two wt strains but not in PPARα‐null mice (Valles et al. 2003). These data strongly suggest that PPARα activation (KE 1) results in gene changes (KE 2) that are responsible for subsequent increases in cell proliferation (KE 3).

It has also been suggested that cell proliferation is mediated by pro‐inflammatory responses (e.g., cytokine secretion) in nonparenchymal liver cells such as Kupffer cells (Corton et al. 2018). Two mouse studies were identified that demonstrated increases in IL‐1β and/or TNF‐α following exposure to ≥ 20 mg/kg/day DINP (Ma et al. 2014; Ore et al. 2022). The cytokines could potentially contribute to cell proliferation following DINP exposure. Although there is no increase in gene markers of cell proliferation in primary hepatocytes treated with DINP or other prototypical PPARα activators (Figure 1), the lack of response is expected, because increased DNA synthesis does not occur in primary hepatocytes alone without nonparenchymal cells (Hasmall et al. 2000; McMullen et al. 2020).

Despite the absence of molecular data (e.g., transcriptomics, RT‐PCR, Western analyses) for alterations in cell growth pathways, the clear evidence for KE 1 and mitogenic cell proliferation (KE 3) below provides convincing evidence for KE 2 in the MOA for DINP‐induced hepatic carcinogenesis in rodents.

### 3.3 KE 3: Perturbation of Cell Growth and Survival

Evidence for the third KE, perturbation of cell growth and survival, includes hepatocellular proliferation and altered apoptosis. Both strong and weak PPARα activators induce hepatocellular proliferation during acute exposures, with strong PPARα activators leading to sustained proliferation during chronic exposure (Corton et al. 2014, 2018; Peters et al. 1998; Ward et al. 1988). Increased cellular DNA synthesis and mitotic figures are indicators of hepatocellular proliferation (Heintz et al. 2023). The MOA for PPARα and CAR‐mediated liver tumorigenesis in rodents shares similar KEs (Yamada et al. 2025). In describing the CAR‐mediated MOA for dieldrin‐induced liver tumors in rodents, DNA synthesis and labeling index are observed largely at short exposure durations (Klaunig and Cohen 2024). Furthermore, even though the initially increased rates of DNA synthesis may decline over time, “the number of hepatocytes has increased as indicated by the increased liver weight, so that the total number of hepatocyte DNA replications is actually continuously increased” (Klaunig and Cohen 2024; Strupp et al. 2023). Thus, direct evidence of continuous cell proliferation is not required because the overall increase in DNA replications from an initial increased cellularity can increase the chance of tumor development. Acute exposures to PPARα activators suppress apoptosis, with potentially increased apoptosis following chronic exposures as a response to the increased cellular proliferation (Corton et al. 2014; Corton et al. 2018). However, as discussed in Yamada et al. (2025), associative events such as decreased apoptosis are not critical for establishing the MOA for PPARα activators. Further, reductions in apoptosis are difficult to detect in vivo due to the inherently low background level (Yamada et al. 2025). Much of the evidence for suppression of apoptosis is based on in vitro studies, and Corton et al. (2018) acknowledge that there is evidence for *increased* apoptosis following longer term in vivo exposure to PPARα activators.

The WoE supports hepatocellular proliferation following acute (1–2 weeks) but not (sub)chronic (13 weeks to 2 years) exposure to DINP in rats and mice. This initial increase in proliferation seen with acute exposure and the lack of such an increase following (sub)chronic exposure is consistent with DINP as a weak PPARα activator (Table 4; additional study details found in Table S2) (Covance 1998a, 1998b; Hazleton 1992 as cited by US EPA 2025; Kaufmann et al. 2002; Smith et al. 2000; Valles et al. 2003). The mixed results in rodents at 4 weeks of DINP exposure are consistent with the transition from acute to (sub)chronic change in the proliferative response. This transition in proliferation was captured in a study of B6C3F1 mice, in which statistically significant increases in hepatocellular proliferation were observed in at least one of three liver zones after 1 week of exposure to DINP in males at ≥ 100 mg/kg/day, and in females at ≥ 800 mg/kg/day, compared to observations at week 4, in which no dose‐responsive hepatocellular proliferation was observed in females, and fewer statistically significant and dose‐dependent proliferation findings were observed in males (Kaufmann et al. 2002). The essentiality of PPARα in this response was demonstrated through the use of PPARα‐null mice, in a study wherein—in contrast to two wt mice strains during acute exposures—female PPARα‐null mice administered DINP for 1 week did not exhibit hepatocellular proliferation (Valles et al. 2003). The in vivo evidence for hepatocellular proliferation in rats and mice is supported further by in vitro evidence in primary rat hepatocytes, in which increased proliferation, as indicated by BrdU, was observed at 250 to 750 μM DINP and ≥ 150 μM MINP (Hasmall et al. 1999; Shaw et al. 2002). Based on these results, it is expected that nonparenchymal cells (especially Kupffer cells), that support hepatocyte proliferation, were present in cultures used in these studies (Hasmall et al. 2000). Hepatocellular proliferation was not observed in primary human hepatocytes at the same concentrations (Hasmall et al. 1999; Shaw et al. 2002), nor was it observed in nonhuman primates administered DINP for 2 weeks at 500 mg/kg/day (Pugh et al. 2000).

**TABLE 4:** Summary of toxicological evidence supporting KE 3 (Perturbation of Cell Growth and Survival) in the PPARα MOA for rodent liver tumors.^a^

	Rodents			Nonhuman primates and humans		
Effect	WoE result	Model	Reference(s)	WoE result	Model	Reference(s)
KE 3: Perturbation of cell growth and survival						
Hepatocellular proliferation	−	Mouse, diet, 4 days	Hazleton (1992) as cited by US EPA (2025)	−	Primary human hepatocytes; DINP	Hasmall et al. (1999)
	+	Rat, diet, 1 week	Covance (1998b)	−	Primary human hepatocytes; MINP	Shaw et al. (2002)
	+	Mouse, diet, 1 week	Kaufmann et al. (2002); Valles et al. (2003)	−	Monkey, gavage, 2 weeks	Pugh et al. (2000)
	−	PPAR‐null mouse, diet, 1 week	Valles et al. (2003)			
	+/−	Rat, diet, 2 weeks	Smith et al. (2000); Covance (1998b)			
	+	Mouse, diet, 2 weeks	Smith et al. (2000)			
	+	Rat, diet, 4 weeks	Smith et al. (2000)			
	+/−	Mouse, diet, 4 weeks	Smith et al. (2000); Kaufmann et al. (2002); Hazleton (1992) as cited by US EPA (2025)			
	−	Rat, diet, 13 weeks	Covance (1998b)			
	−	Mouse, diet, 13 weeks	Hazleton (1992) as cited by US EPA (2025)			
	−	Mouse, diet 78/79 weeks	Covance (1998a)			
	−	Rat, diet, 2 years	Covance (1998b)			
	−	Mouse, diet, 2 years	Covance (1998a)			
	+	Primary rat hepatocytes; DINP	Hasmall et al. (1999)			
	+	Primary rat hepatocytes; MINP	Shaw et al. (2002)			
Apoptosis	+/−	Mouse, diet, 1 week	Kaufmann et al. (2002)	−	Primary human hepatocytes; DINP	Hasmall et al. (1999)
	−	Mouse, diet, 4 weeks	Kaufmann et al. 2002	−	Primary human hepatocytes; MINP	Shaw et al. 2002
	+	Primary rat hepatocytes; DINP	Hasmall et al. (1999)			
	+	Primary rat hepatocytes; MINP	Shaw et al. (2002)			

^a^ A more detailed summary of these studies is provided in Table S2.

Limited and mixed data are available regarding alterations to apoptosis following DINP treatment (Hasmall et al. 1999; Kaufmann et al. 2002; Shaw et al. 2002). In the single in vivo study in B6C3F1 mice administered DINP in the diet for 1 and 4 weeks, non–dose‐dependent changes in apoptosis were observed at 1 week, and no effect on apoptosis was observed at 4 weeks (Kaufmann et al. 2002). In vitro, decreased apoptosis findings were consistent with the aforementioned increases in hepatocellular proliferation observed in primary rat but not human hepatocytes. In primary rat hepatocytes, decreased spontaneous apoptosis was observed at ≥ 250 DINP and decreased TGF‐β1‐induced apoptosis was observed at ≥ 500 μM DINP (Hasmall et al. 1999). Further, decreased TGF‐β1‐induced apoptosis was observed at ≥ 150 μM MINP (Shaw et al. 2002). No changes in apoptosis were observed in primary human hepatocytes treated with the same concentrations of DINP or MINP (Hasmall et al. 1999; Shaw et al. 2002). However, as noted above, in vitro and in vivo studies of PPARα activators do not consistently show decreased apoptosis, and establishing decreased apoptosis is not critical for establishing the PPARα MOA for liver tumors (Corton et al. 2018; Yamada et al. 2025).

As discussed for KE 1, increases in absolute or relative liver weight can indicate hepatocellular hyperplasia (KE 3) or hepatocellular hypertrophy (KE 1) or both. Collectively, the WoE indicates that DINP is a weak PPARα activator that induces hepatocellular proliferation from short‐term exposures but does not induce sustained proliferation from (sub)chronic exposures. The dataset from PPARα‐null mice corroborates the finding that hepatocyte proliferation is PPARα‐dependent (Valles et al. 2003).

### 3.4 KE 4: Clonal Expansion of Preneoplastic Foci

The fourth KE in the PPARα MOA for liver tumors is clonal expansion of preneoplastic foci (Corton et al. 2014, 2018). Preneoplastic foci are areas of local hepatocyte proliferation or hyperplasia and can also be referred to as foci of cellular alteration, areas of cellular alteration, foci of altered hepatocytes, hyperplastic foci, enzyme‐altered foci, or phenotypically altered foci (Thoolen et al. 2010). These are often characterized as foci of basophilic and/or eosinophilic cells (Thoolen et al. 2010). Evidence for this KE in the PPARα MOA largely includes basophilic foci that lack glutathione S‐transferase placental form or γ‐glutamyl transpeptidase, selective increase in DNA replication in the foci, increased apoptosis in the foci, and a reduction of cells in the foci after cessation of exposure to the peroxisome proliferator (Corton et al. 2014).

Only a minimal body of data are available to support KE 4. In female mice exposed to DINP in the diet at doses equivalent to 0, 112, 336, 910, or 1888 mg/kg/day, the incidence of “cellular alteration, basophilic” among all deaths was 0/70, 0/61, 0/60, 1/60, and 3/70, respectively (Covance 1998a). Incidences in males exposed to 0, 90, 276, 742, or 1560 mg/kg/day were more variable at 1/70, 1/60, 2/60, 0/60, 2/70, respectively. Among the rat studies in which significant increases in liver tumors were observed, cellular alterations (basophilic or eosinophilic) tended to decrease with dose (Biodynamics 1987; Covance 1998b). In Covance (1998b), this pattern of decreased incidence of cellular alterations was observed at both the interim (79 weeks) and 2‐year timepoints, with high incidences of basophilic cellular alteration in control males (4/15) and females (7/15) at 79 weeks and in control males (34/41) and females (33/42) at 2 years. Similar results were reported for eosinophilic cellular alterations. This pattern was also observed in the F344 rat study wherein doses were not sufficient to induce liver tumors (Lington et al. 1997). Therein, the incidences of both basophilic and eosinophilic foci were high in the controls of both sexes (e.g., 72/81 basophilic foci in females) and decreased slightly with dose. The slight reduction in foci at study termination is unlikely explained by progression to tumors, because liver tumors were not increased significantly in Lington et al. (1997). These findings suggest that altered foci may have limited utility for informing the PPARα MOA in rats, at least in the available DINP studies.

Further, as discussed in Yamada et al. (2025), altered hepatic foci may not be observed in some studies, because, *“*[d]epending on the time points selected, increases in altered hepatic foci may not be observed and at the terminal necropsy the presence of large liver tumors may make it difficult to quantify numbers of altered hepatic foci. However, as altered hepatic foci are considered to be the precursor lesion for subsequent tumor formation (Thoolen et al. 2010), the absence of such data does not detract from establishing a robust MOA.” As an example, PPARα activator HFPO‐DA significantly increased liver tumors in male and female mice, yet the incidences of basophilic foci were increased significantly only in males at study termination, despite evidence for a PPARα MOA in both sexes (Heintz et al. 2023; Thompson et al. 2025). In the rat DINP studies, the background incidence of foci complicates the establishment of evidence for KE 4. Nevertheless, the strong evidence for KE 3 and the AO (i.e., liver tumors) suggests that the clonal expansion occurred in the DINP studies.

### 3.5 Modulating Factors

In addition to the DINP evidence for the KEs in the PPARα MOA for liver tumors in rodents, there is supporting evidence for modulating factors. Unlike KEs, the modulating factors of the PPARα MOA for liver tumors in rodents may be PPARα dependent or independent, and although their role does not rise to the criteria of a KE, they may modify (i.e., enhance or inhibit) other KEs in the pathway (Corton et al. 2014). These modulating factors include oxidative stress, nuclear factor kappa B (NF‐kB) activation, and reduced gap junction intercellular communication (GJIC). Oxidative stress can result in DNA damage and contribute to liver tumor promotion from PPARα activators, which themselves do not induce DNA damage and are nongenotoxic (see Section 5.1) (Corton et al. 2014). NF‐kB, which is involved in immune responses and cancer progression, is activated under oxidative stress conditions and may be the result of Kupffer cell production of cytokines (KE 2) (Corton et al. 2014). Reduced GJIC, representing a protentional perturbation in tissue homeostasis, has been considered a modulating factor of cell proliferation (KE 3) (Corton et al. 2014; Yamada et al. 2025) and a nongenotoxic mechanism of carcinogenesis (Chipman et al. 2003). The evidence related to each of these modulating factors is explored in rats and mice, as well as humans and nonhuman primates.

The overall DINP evidence from in vivo and in vitro rodent studies supports hepatic oxidative stress and reduced GJIC. However, no data were identified regarding hepatic NF‐kB activation in rodents. Various indicators of oxidative stress were observed in the livers of mice orally administered DINP at ≥ 20 mg/kg/day for 2–4 weeks: increased reactive oxygen species (ROS), decreased glutathione (GSH), increased GSH peroxidase and catalase (CAT) activity, increased lipid peroxidation, increased cytokine production (see KE 2), and DNA damage (Liang and Yan 2020; Ma et al. 2014; Ore et al. 2022). Both mice and rats administered dietary DINP for 2 and 4 weeks exhibited reduced GJIC in the liver (Smith et al. 2000), and reduced GJIC was observed in rat liver oval cells treated with DINP in vitro (Ctverackova et al. 2020). Furthermore, three MINP isomers inhibited GJIC in rat and mouse hepatocytes in a concentration‐dependent manner (Kamendulis et al. 2002).

Compared to rodents, the minimal evidence for these modulating factors was mixed in nonhuman primates, humans, and human cell lines. Across epidemiological investigations examining urinary biomarkers related to oxidative stress, there was no evidence of oxidative DNA damage (Ketema et al. 2022; Zhang et al. 2021), and mixed evidence related to lipid peroxidation was found (Ketema et al. 2022; Ortlund et al. 2024). Increased ROS was reported in HEPG2 cells (Gutierrez‐Garcia et al. 2019); however, cell culture conditions can promote ROS elevation that is not necessarily indicative of in vivo responses (IARC 2025). No reduction or inhibition in GJIC was observed in vivo or in vitro based on studies in nonhuman primates given DINP at 500 mg/kg/day (Pugh et al. 2000) and in hamster, cynomolgus, or human hepatocytes treated with three MINP isomers (Kamendulis et al. 2002), respectively. NF‐kB activity was not examined in vitro in any human liver cell lines; however, DINP increased NF‐kB activity in the human kidney cell line HEK293 (Tas et al. 2019).

Collectively, the available data provide supporting evidence for oxidative stress and reduced GJIC in rodents, while the limited data set in humans is mixed regarding oxidative stress but generally does not support DINP‐reduced GJIC in humans.

## 4 Modified Bradford Hill Criteria

In accordance with the MOA and human relevance frameworks built from IPCS and the US EPA Guidelines for Carcinogen Risk Assessment, the Modified Bradford Hill Criteria are applied to assess the evidence identified for DINP and the PPARα MOA for liver tumors in rodents (Meek et al. 2014; US EPA 2005). The Modified Bradford Hill Criteria include biological concordance, essentiality, dose and temporal concordance, consistency, and analogy. The strength and consistency of the evidence for KEs 1–4 are described in the MOA evaluation and demonstrate that DINP induces rat and mouse liver tumors through the PPARα MOA; however, it does not support a conclusion that this MOA operates in nonhuman primates and humans, consistent with differences in PPARα‐regulated gene networks between species.

Biological concordance and analogy support a finding that DINP acts through the PPARα MOA for liver tumors in rodents. This MOA for rodent liver tumors is well‐established for a wide range of chemical classes, including fibrate drugs, some per‐ and polyfluoroalkyl substances (PFAS), and phthalates and phthalate metabolites (Corton et al. 2014, 2018; Felter et al. 2018; Yamada et al. 2025). Specifically, diethylhexyl phthalate (DEHP), di‐n‐butyl phthalate (DBP), and DEHP's metabolite monoethylhexyl phthalate (MEHP) are known PPARα activators associated with liver tumors in rodents (Corton et al. 2014, 2018; Corton and Lapinskas 2005; De Abrew et al. 2015; Lapinskas et al. 2005; Ren et al. 2010; Yamada et al. 2025). As such, it is anticipated that DINP and its monoester metabolite MINP would act through a PPARα MOA. The evidence presented in support of DINP and MINP acting as PPARα activators (including associative events), leading to altered cell growth and proliferation in rats and mice, is consistent with a well‐established MOA for liver tumors in rodents, an MOA through which other phthalates are known to act.

Evidence for essentiality comes from preventing KEs or observing reversibility of effects after cessation of exposure (Meek et al. 2014). The wt and PPARα‐null mouse model experiments by Valles et al. (2003) clearly demonstrated that the increased PCO and ACO activity/expression and hepatocyte proliferation observed following 1 and/or 3 weeks of DINP exposure were dependent on KE 1, PPARα activation. Further, the WoE across these PPARα‐null mice indicates that increased liver weight is PPARα‐dependent (Valles et al. 2003), with potential for compensatory CAR‐dependent increases in liver weight in PPARα‐null mice, consistent with observations in PPARα‐null rodents treated with a known PPARα activator DEHP (Corton et al. 2018; Ito et al. 2007). Notably, the CAR MOA for liver tumors is generally recognized as not relevant to humans (see Section 5.4) (Elcombe et al. 2014; Felter et al. 2018; Shizu et al. 2024; Yamada et al. 2025). Additional evidence supporting the essentiality of PPARα in the rodent tumor response to DINP includes less pronounced evidence for KEs 1 and 3 in mice exposed to DINP with a recovery period (Covance 1998a). In this study, B6C3F1 mice were administered DINP at up to 8000 ppm for 2 years, with a recovery group exposed to DINP at 8000 ppm for 78 weeks followed by a 26‐week recovery period (Covance 1998a). The incidences of both hepatocellular enlargement and cytoplasmic eosinophilia and the magnitude of increased liver weight in males were less pronounced in the recovery group (Covance 1998a). In a chronic bioassay in F344 rats administered DINP at up to 12,000 ppm for 2 years or at 12,000 ppm for 78 weeks followed by a 26‐week recovery period, liver weight was increased at 104 weeks but not in the recovery group (Covance 1998b). Collectively, the rodent DINP data support the essentiality of PPARα in the rodent liver tumor MOA after DINP exposure.

From a temporal perspective, DINP induces PPARα activation and alteration of cell growth (cell proliferation) and survival within just a few days/weeks of exposure. Peroxisomal enzyme activity and hepatocellular hypertrophy are observed across study durations, supporting chronic activation. Liver tumors have generally not been observed at interim timepoints, suggesting that DINP is a weak PPARα carcinogen in rodents that requires prolonged exposure to induce a modest increase in tumor incidence in rats and a stronger response in mice. Dose concordance and temporality data in rats indicate PPARα activation (KE 1) as early as 1 week and at concentrations of ≥ 1000 ppm, as well as perturbation of cell growth and survival (KE 3) as early as 4 days and at concentrations as low as 2500 ppm and liver tumors at 2 years with doses of ≥ 10,000 ppm in rats (Figure 2). Although some limitations in the DINP database, including study designs with limited doses and KEs evaluated (i.e., paucity of KE 2 data), preclude a complete depiction of the dose‐ and temporal‐concordance, the data are nonetheless compelling. It should also be recognized that a complete picture of an MOA may not be feasible for weaker‐acting chemicals that do not have a substantial number of molecular studies or studies with ideal dose spacing. As such, studies that use earlier time points (hours to days) and smaller dose spacing than existing assessments could improve dose and temporal concordance. It is also conceivable that dose and temporal separation may not be readily achieved. For example, given homeostatic mechanisms within cells and tissues (Stanger 2015), the early KEs may occur nearly concurrently, with only subtle differences in timing. The absence of such studies is not considered evidence against temporal or dose concordance, and the existing database does broadly support both.

**FIGURE 2:**
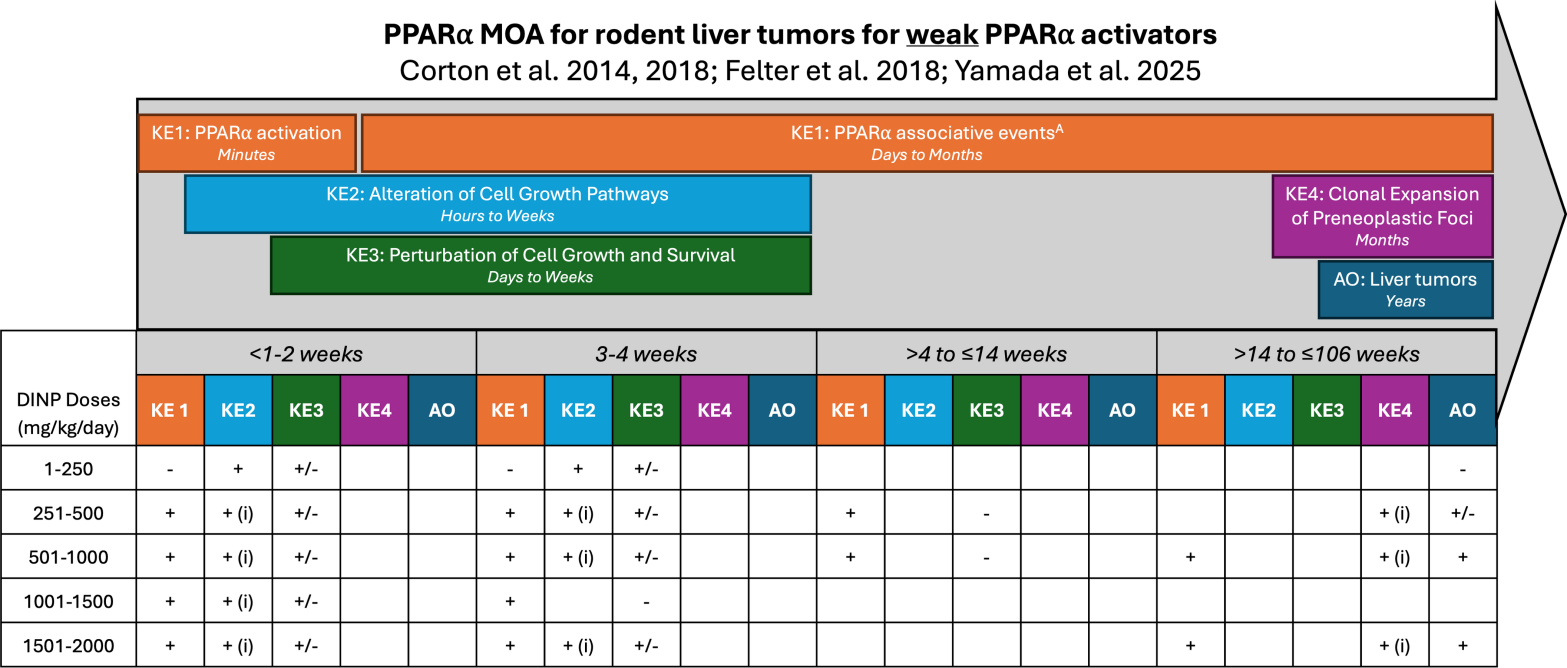
Dose and temporal concordance evidence for the PPARa MOA for liver tumors in mice. + indicates DINP exposure led to observation of KE at specified timpoint;‐indicates DINP did not lead to observation of KE at specified timepoint;+/− indicated mixed results for KE at timepoint across studies; (i) indicates the KE is inferred based on strong evidence for KE before and after; blank cell indicates no data for KE at specified timepoint ^A^Includes PBOX,PCO/ACO activity, peroxisome proliferation, hepatocellular, and increased liver weight.

The WoE evaluated according to the Modified Bradford Hill Criteria supports that DINP acts through the PPARα MOA to induce liver tumors in rats and mice.

## 5 Alternative MOAs

There are three primary mechanisms of carcinogenesis: DNA damage, immunosuppression, and increased stem cell replication (Cohen et al. 2019). Cancers related to increased stem cell replication can occur through mitogenesis (e.g., endocrine disruption or nuclear receptor activation) or cytotoxicity‐induced regenerative cell proliferation. Cancers related to immunosuppression are usually the result of viral disorders in humans and potentially reduced tumor immunosurveillance; however, data indicate that chemicals that induce immunosuppression are rarely positive in animal cancer bioassays and rodent cancer bioassays may not reliably detect cancer risk from immunomodulatory agents (Bugelski et al. 2010; Cohen et al. 2019; Lebrec et al. 2016). As discussed above, there is ample evidence for a nuclear receptor‐mediated mitogenesis MOA for DINP involving PPARα. Evidence for MOAs that involve DNA damage, cytotoxicity, endocrine disruption, and immunosuppression are addressed below.

### 5.1 DNA Damage

Peroxisome proliferators are broadly considered to be nongenotoxic (IARC 1995). The systematic evaluation of the publicly available DINP‐specific evidence supports a lack of genotoxicity of DINP (Lea et al. In Press) and is consistent with authoritative and agency conclusions that DINP is nongenotoxic and/or nonmutagenic (ECHA 2013; HC 2015; IARC 1995; OEHHA 2013; US EPA 2025a). DINP has been found to be nonmutagenic in a variety of in vitro and in vivo assays by several research groups (Barber et al. 2000; McKee et al. 2000; Zeiger et al. 1985). The reader is referred to Table 1‐1 in US EPA (2025a) for a comprehensive summary of DINP genotoxicity studies.

### 5.2 Endocrine Disruption

A comprehensive evaluation of the endocrine‐disrupting potential of DINP found that DINP does not meet the ECHA/EFSA criteria to be considered an endocrine disruptor (Lea et al. 2025). Further, the role of receptor‐mediated effects, including androgen, estrogen, and thyroid receptors, in DINP carcinogenesis was systematically evaluated by Lea et al. (In Press), and it was found that in vivo and in vitro evidence does not support an estrogen or thyroid MOA for DINP‐induced tumors, and only weak evidence was reported for the androgen receptor (AR), which was not specific to the liver. Namely, human AR antagonist activity was observed in two out of six in vitro assays, and increased testosterone was reported in one of two H295R steroidogenesis assays (Lea et al. In Press). These findings are consistent with the lack of active ToxCast assays reported by US EPA (2025a). Thus, there is no evidence to support an endocrine‐mediated (androgen, estrogen, thyroid) MOA for liver tumors resulting from DINP exposure.

### 5.3 Immunosuppression

US EPA (2025d) evaluated the potential for DINP to cause immunotoxicity in their evaluation of noncancer effects. No data were deemed sufficient to carry forward in their dose–response assessment, indicating that the MOA for DINP‐induced liver tumors is not mediated by an MOA involving immunosuppression. Further, the systematic review of the evidence related to the potential carcinogenicity of DINP found only inconsistent evidence related to immunosuppression, with positive data limited to some changes in hematological parameters in rodents and nonhuman primates, and a single in vitro assay demonstrating decreased phagocytosis (Lea et al. In Press).

### 5.4 Non‐PPARα Nuclear Receptors

Among PPAR isoforms, PPARα is expressed predominantly in the liver, whereas PPARγ and PPARβ have low hepatic expression, with PPARγ expressed predominantly in adipose tissues and PPARβ expressed in numerous tissues, including the skin, muscle, adipose, and heart (Chawla et al. 1994; Corrales et al. 2018; Wagner and Wagner 2010). The ability of DINP and/or MINP to activate PPARγ and/or PPARβ has been examined through reporter gene assays (Bility et al. 2004; Fang et al. 2015; Laurenzana et al. 2016; Pomatto et al. 2018; Schaffert et al. 2022). Bility et al. (2004) showed that MINP activated mouse PPARα and PPARγ at similar concentrations, yet the maximum fold change of PPARα activation (27.1 ± 1.9) was nearly twice the maximum fold change PPARγ activation (14.1 ± 1.0). For human PPAR, MINP activated PPARα at lower concentrations than PPARγ; however, the maximum fold change was higher for human PPARγ (9.3 ± 1.2) than human PPARα (5.8 ± 2.1) (Bility et al. 2004). In contrast, Laurenzana et al. (2016) reported that MINP activates human PPARα, PPARβ, and PPARγ isoforms at a similar concentration and with similar magnitudes of response. In a reporter gene assay, no hPPARγ activation was observed in HEK293 cells at up to 400 μM DINP (Schaffert et al. 2022). Dose‐responsive increases in PPARγ activity were observed in two other reporter gene assays at DINP concentrations up to 33 μM (Fang et al. 2015; Pomatto et al. 2018); PPARα and PPARβ activation was not assessed in these studies. US EPA ToxCast DINP bioactivity data concerning human PPARγ and PPARβ show no activity for neither agonism nor antagonism (US EPA 2025b, 2025c). However, given the rapid metabolism of DINP in vivo, the relevance of these assays is questionable.

CAR and pregnane x receptor (PXR) are xenobiotic sensing receptors that regulate expression of xenobiotic‐metabolizing enzymes (OEHHA 2013; Ren et al. 2010). Some peroxisome proliferators have been known to activate CAR and PXR (Ren et al. 2010). CAR activation is a mechanism by which some liver tumors form in rodents, which, like PPARα activators, are widely considered to lack human relevance (Felter et al. 2018; Shizu et al. 2024; Yamada et al. 2025). Similarly, Elcombe et al. (2014) reported that the CAR MOA for liver tumors is “qualitatively not plausible in humans.” Evidence for the lack of human relevance regarding the CAR MOA for liver tumors is based on data demonstrating (1) that compounds which act through CAR to elicit liver tumors do not elicit tumors in CAR‐null rodents, (2) a lack of replicative DNA synthesis in CAR‐null rodents, human hepatocytes, and a chimeric mouse/human model when treated with CAR activators, and (3) that there is a lack of epidemiological findings related to cancer in humans following chronic exposure to phenobarbital (a known CAR agonist) (Felter et al. 2018). Most recently, Yamada et al. (2025) concluded that “the time has come to consider that chemicals for which the MOA of liver carcinogenicity observed in rodents has been clearly identified as either CAR‐ or PPARα‐mediated (with other MOAs having been excluded) can be judged as having no relevance to human cancer risk without further investigations being necessary.”

As shown in Figure 1, transcriptomic profiling is conducted in rat primary hepatocytes exposed to multiple chemicals (each at two doses: “mid” [dose 1; D1] and “very high” [D2]) with different known MOAs (e.g., PPARα, CAR, and other receptor agonists) for 24 h (time 1; T1) and 48 h (T2). At T1, the transcriptomic profiles from D2 DINP (500 mM) and another phthalate, DEHP (500 mM), clustered with profiles from D2 concentrations of WY14643 (40 mM) and clofibrate (300 mM), both known PPARα activators, but did not cluster with the profile of D2 phenobarbital (1000 mM), the known CAR agonist (De Abrew et al. 2015). This suggests a largely PPARα‐dependent MOA for DINP and DEHP. However, it should be noted that the MOA‐based clustering analysis did not produce chemical groups based on toxicity class for the other dose and time combinations (T1 x D1; T2 x D1; T2 x D2), and the authors highlight the importance of high‐dose selection and appropriate timepoint selection in in vitro MOA assessments (De Abrew et al. 2015). No in vivo studies were identified that examined the CAR/PXR MOA for induction of liver tumors in rodents by DINP.

In vitro data provide some evidence of CAR and PXR activation by DINP. For example, a transactivation assay of COS‐1 cells transfected with hCAR isoforms or hPXR treated with 5a‐androstan‐3a‐ol followed by DINP activated hCAR2 at ≥ 0.1 μM and hPXR at 3 μM but did not activate hCAR1 or hCAR3 at any concentration up to 10 μM (DeKeyser et al. 2011). Similar results were observed in a two‐hybrid protein interaction assay (DeKeyser et al. 2011). Another transactivation assay study evaluated the potential for MINP (up to 100 μM) and DINP (1 μM only) to activate hCAR1, hCAR2, hCAR3, and hPXR following 5a‐androstan‐3a‐ol treatment (Laurenzana et al. 2016). MINP activated hCAR2 at ≥ 1 μM and hPXR at ≥ 10 μM; no statistically significant activation was observed for hCAR1 or hCAR3 (Laurenzana et al. 2016). DINP only activated hCAR2 and hPXR significantly above control (Laurenzana et al. 2016). Together, these studies demonstrate that MINP and DINP activate hCAR2 and hPXR in vitro (DeKeyser et al. 2011; Laurenzana et al. 2016). The hCAR2 splice variant is not conserved across rats, mice, or monkeys (DeKeyser et al. 2011); therefore, activation of this splice variant is not involved in the development of rodent liver tumors.

Activation of other receptors, such as the aryl hydrocarbon receptor (AhR), has been proposed as potential mechanisms through which liver tumors could develop, but relevant DINP data have been negative. A transactivation assay with AhR was conducted in hepa1.12cR cells at concentrations of up to 10^−4^ M DINP, without any significant effects (Kruger et al. 2008). DINP was also inactive in assays for AhR and CAR through US EPA's ToxCast program (US EPA 2025b, 2025c). Again, however, the rapid metabolism of DINP in vivo makes the relevance of these assays uncertain.

### 5.5 Cytotoxicity and Regenerative Cell Proliferation

Criteria that have been proposed for establishing a cytotoxicity MOA include (1) the chemical is not DNA reactive, (2) there is clear evidence of cytotoxicity via histopathology, (3) toxicity is supported by presence of increased serum enzymes that are relevant to humans, (4) there is increased cell proliferation or number of hepatocytes, (5) the dose–response curves for cellular proliferation and the formation of tumors are parallel, and (6) cytotoxicity is reversible (ideally, although not required) (Felter et al. 2018). Carbon tetrachloride is a chemical with a proposed cytotoxic MOA, as evidenced by concordance between liver tumors and liver fibrosis and cirrhosis (indicative of regenerative responses) in rats following inhalation exposure (Cohen et al. 2023; Nagano et al. 2007). In the context of animal bioassays, fibrosis and cirrhosis are indicative of prolonged regenerative hyperplastic responses (Greaves 2012; Thoolen et al. 2010). Importantly, no such lesions were reported in the chronic DINP studies. Furthermore, several of the chronic animal bioassays included terminal and interim assessments of BrdU labeling index (measure of cell proliferation) in the liver, as well as measurement of PCO (indicator of peroxisomal proliferation) in the liver. In mice, ~10‐fold increases in PCO activity were observed in the highest dose groups of both sexes, whereas no significant differences in labeling index were observed in high‐dose mice compared to controls (Covance 1998a). In rats, significant increases in PCO activity were observed in the highest dose groups of both sexes at 1, 2, 13, and 104 weeks of exposure, whereas significant increases in LI were observed only at 1 week of exposure (Covance 1998b). These data do not provide evidence for prolonged cell proliferation in response to ongoing cytotoxic injury.

Focal necrosis has been reported in studies conducted in rats. Lington et al. (1997) reported focal necrosis in male and female rats at 2 years but not at an 18‐month interim sacrifice. At 2 years, there was also an increase in spongiosis hepatis, which is thought to be idiosyncratic to rats (Greaves 2012). A similar pattern was observed in SD rats (Biodynamics 1987). Spongiosis hepatis is often seen in or associated with necrosis, cellular enlargement, and neoplasms (Thoolen et al. 2010). Notably, neither focal necrosis nor spongiosis hepatis was observed in mice exposed to DINP for up to 2 years (Covance 1998a). Taken together—the specificity in rats and the absence of regenerative lesions (see above)—the observed necrosis is unlikely indicative of a cytotoxic MOA.

Increases in serum liver enzymes (e.g., ALT, AST, and ALP) can indicate liver toxicity; however, it is generally accepted that the level of fold changes is more relevant than statistical significance alone. Exact fold changes indicative of toxicity or concern are not specified, but exceedance of ALT twofold to fourfold has been proposed (Hall et al. 1999). However, there is increasing evidence that elevated serum ALP may be the result of PPARα‐mediated increases in *Alpl* gene expression (Corton et al. 2020; Heintz et al. 2023, Heintz et al. 2025; Thompson et al. 2025; Vallanat et al. 2010). Increases in serum liver enzymes in Lington et al. (1997) rarely exceeded threefold and occurred at earlier timepoints in concert with hepatocellular hypertrophy but where focal necrosis was not observed. Increases in serum liver enzymes have been reported in DINP chronic bioassays; however, the lack of histopathological evidence for a cytotoxicity MOA renders their relevance to MOA analysis uncertain. Notably, US EPA (2025a) concluded that the “available data do not consistently support the various KEs in the MOA for cytotoxicity, suggesting other MOAs are at play.”

## 6 Human Relevance

Human relevance addresses three fundamental questions (Boobis et al. 2006, 2008; Meek et al. 2003):

1. Is the WoE sufficient to establish the MOA in animals?
2. Are the KEs plausible in humans, or are there fundamental qualitative differences in KEs between humans and rodents?
3. Are the KEs plausible in humans after accounting for quantitative pharmacokinetic and pharmacodynamic differences between humans and rodents?

Regarding Question 1, based on findings summarized herein, the WoE is sufficient to establish that the MOA for liver effects in rodents exposed to DINP involves PPARα. IARC (1995) reported that evidence from hypolipidemic agents used in humans indicates that they produce “little if any peroxisome proliferation”; however, IARC noted that compounds that elicit peroxisome proliferation in rodents should be evaluated on a case‐by‐case basis for carcinogenicity in humans. Therefore, to answer Question 2, general biological information about rodent and human PPARα and DINP‐specific evidence is considered. Both streams of evidence support that there are fundamental qualitative differences in KEs that preclude PPARα‐dependent liver tumor formation in humans. Specifically, KE 2 and KE 3 (i.e., changes in gene expression related to cell cycle and alteration in cell growth and survival) do not occur in nonhuman primates and humans, and, as such, downstream events are absent (Corton et al. 2014, 2018; Felter et al. 2018). This is further supported by the fact that elevated risk of liver cancer (the AO) in humans has not been associated with chronic exposure to the PPARα activators, gemfibrozil, and clofibrate (Corton et al. 2014; Peters et al. 2005).

The body of data regarding the biological differences between humans and rodents that support the lack of human relevance of the PPARα liver tumor MOA includes the following: (1) decreased PPARα expression in humans compared to rodents, (2) decreased sensitivity to ligands of hPPARα relative to rodent PPARα, (3) divergent downstream transcriptional networks controlled by hPPARα relative to rodent PPARα, (4) differences in specificity of PPARα for the genomic response element sequence (peroxisome proliferator‐activated response element—PPRE) in rodents and humans, and (5) decreased sensitivity of humanized PPARα mice to GW7647, a potent PPARα agonist, compared to wt mice. These biological differences are explored herein.

Several reviews have concluded that humans and nonhuman primates are less responsive to PPARα activators, due to a combination of differences in PPARα expression, PPARα isoforms, and differences in target gene expression following PPARα activation (Corton et al. 2014; HC 2015; Yamada et al. 2025). Information on relative expression between rodents and humans is mixed, with some reporting either decreased or no differences in expression in humans relative to rodents (Palmer et al. 1998; Rakhshandehroo et al. 2009). A truncated form of PPARα that lacks the hinge region and the ligand binding domain of functional PPARα was found to constitute a significant portion of the hPPARα mRNA (10%–50%) but less than 10% of PPARα in mice (Corton et al. 2014; Palmer et al. 1998). Transactivation assays with human and rodent PPARα constructs exposed to PPARα agonists such as WY14643, clofibrate, perfluorooctanoic acid (PFOA), and phthalates demonstrate differences in sensitivity that are speculated to be due to structural differences in the PPARα ligand binding domain or structural differences in the promoter regions of the target genes (Corton et al. 2014).

PPARα regulates transcriptional networks related to lipid metabolism in both rodents and primates; however, only rodent PPARα regulates transcriptional networks related to cellular proliferation (as reviewed by Corton et al. 2014; Felter et al. 2018; McMullen et al. 2014, 2020; Rakhshandehroo et al. 2009). McMullen et al. (2020) found that, at low doses, the transcriptional responses were similar between rat and human hepatocytes exposed to the potent hPPARα agonist GW7647 (including upregulation of lipid and metabolism pathways), but at high doses, upregulation of genes related to the cell cycle, apoptosis, and cytoskeletal reorganization was observed only in rat hepatocytes. Similar results have been observed in rodent and human hepatocytes exposed to HFPO‐DA (Heintz et al. 2024), which causes liver tumors in mice via a PPARα MOA (Heintz et al. 2023, 2024; Thompson et al. 2025). In addition to the different transcriptional networks, the sequence and structure of the PPRE is not conserved between rats and humans (McMullen et al. 2020).

These differences between rodents and humans are also observed in humanized PPARα mice treated with WY14643, where, unlike wt mice, there was no activation in genes involved in cellular proliferation (Yang et al. 2008). Although hepatocyte proliferation was observed in humanized mice, it should be appreciated that hPPARα in humanized mice functions in the presence of host‐mouse cellular machinery and PPREs, and thus, these studies do not demonstrate the ability of hPPARα to elicit transcriptional changes associated with cellular proliferation in humans.

As shown throughout the previous MOA sections and summarized in Table 5, DINP‐specific data are consistent with the general evidence from PPARα activators, such that KEs 2–4 and the AO are not observed following exposure to DINP in humans and/or nonhuman primates. DINP‐specific data supporting a lack of human relevance of the PPARα MOA for liver tumors include the following: (1) decreased sensitivity of hPPARα to MINP relative to rodent PPARα, (2) lack of evidence for β‐oxidation and associated enzyme activity in human and nonhuman primate hepatocytes following DINP or MINP treatment, and (3) lack of perturbation of cell growth and survival in human or nonhuman primate hepatocytes exposed to DINP. These findings are summarized in Tables 3 and 4. In addition, the conclusion on the lack of relevance of the rodent PPARα MOA to humans is supported by the lack of evidence that DINP causes liver tumors in humans in epidemiological studies.

**TABLE 5:** Summary concordance table for DINP PPARα MOA for liver tumors.

Key event	Rats	Mice	Monkeys	Humans
KE 1: PPARα activation	Yes	Yes	Likely; based on evidence for hPPARα activation in human hepatocytes	Yes
KE 2: Altered gene expression	Inferred	Inferred	No; qualitative differences in gene expression related to cell cycle between rodents and primates	No; qualitative differences in gene expression related to cell cycle between rodents and primates
KE 3: Increase in cell proliferation	Yes	Yes	No; data indicate lack of increased cell proliferation in monkey studies	No; data indicate lack of increased cell proliferation in monkey studies and therefore similar results are anticipated in humans. In vitro data in primary human hepatocytes suggest lack of proliferation in model capable of proliferation (as observed with primary rodent cells)
KE 4: Clonal expansion	Limited	Limited	Unlikely; lack of measured data for K E4 but data indicate lack of increased cell proliferation (KE 3) in monkey studies	Unlikely; data indicate lack of increased cell proliferation in monkey studies and therefore similar results are anticipated in humans
AO: Adenomas &carcinomas	Yes	Yes	Unlikely; lack of measured data for AO but data indicate lack of increased cell proliferation (KE 3) in monkey studies	Unlikely; data indicate lack of increased cell proliferation in primates and therefore similar results are anticipated in humans; No epidemiological studies indicating an association between liver tumors and DINP exposure or PPARα activators gemfibrozil and clofibrate

Given the qualitative differences between species in the transcriptional networks controlled by PPARα that prevent KEs subsequent to KE 1 and preclude liver tumor formation in humans and nonhuman primates, Question 3 about quantitative differences does not need to be addressed. DINP‐specific data also support that KE 2–KE 4 and the AO do not occur in humans and/or nonhuman primates following DINP exposure. Overall, there is robust evidence that the MOA for DINP‐induced liver tumors in rodents lacks human relevance.

## 7 Uncertainties and Limitations

The overall WoE presented herein provides strong support for a PPARα MOA for rodent liver tumors following DINP exposure. The evidence is strongest for KEs 1 and 3. Evidence for KE 2 could be strengthened with the addition of in vitro or in vivo transcriptomic analyses. Although there is clear evidence for activation of PPARα target genes in vitro (Figure 1), the available database is insufficient to demonstrate in vivo changes in gene expression related to cell death, proliferation, death, and survival; however, it is reasonable to infer that these changes occur, given the strong evidence for KE 1 and KE 3. The limitations in the design of existing studies (i.e., few early time points, number of doses, and dose spacing) limit these studies' ability to establish a strong temporal and dose concordance, although there is no evidence against these criteria in the current dataset. Additional counterfactual data could strengthen the MOA by demonstrating that removal of PPARα mitigates or prevents all KEs and the AO in the proposed MOA. There is some existing evidence to demonstrate the dependence of DINP‐mediated liver effects on PPARα. Specifically, PPARα‐null mice exposed to DINP (for either 1 or 3 weeks by dietary feed or gavage) did not exhibit increases in β‐oxidation, DNA repair and recombination, or hepatocyte proliferation (Valles et al. 2003). Although increased liver weight was noted in female PPARα‐null mice in one of the four study conditions—specifically, in mice exposed to 8000 ppm DINP in diet for 3 weeks (Valles et al. 2003)—this evidence may be incidental or suggest compensatory actions that have been observed in other studies of PPARα‐null rodents (Corton et al. 2018; Ito et al. 2007).

While US EPA (2025a) concluded that DINP acted through a PPARα MOA, they highlighted limited evidence for inhibition of apoptosis as an uncertainty in the MOA. However, as discussed in Yamada et al. (2025), apoptosis is difficult to quantify in the rodent liver, due to low basal levels. It was also speculated that such inhibition might be more relevant within altered foci (i.e., KE 4), for which it is already acknowledged there are few DINP‐specific data. Furthermore, Corton et al. (2018) acknowledge that increased apoptosis has been observed in in vivo studies of some PPARα activators.

## 8 Conclusion

Several agencies, including US EPA, have concluded that the WoE supports a finding that DINP liver tumors in rodents are the result of a PPARα MOA (ECHA 2013; HC 2015; US EPA 2025a). Herein, a thorough and independent MOA assessment for DINP‐induced rodent liver tumors was conducted through application of principles from key MOA evaluation frameworks (Boobis et al. 2006; Meek et al. 2014; US EPA 2005). The WoE strongly indicates that the liver tumors observed in rodents exposed to DINP occur through a PPARα MOA. The strength of evidence for KE 1 and KE 3 is greater than for KE 2 and KE 4; however, KE 2 and KE 4 are each supported by robust KEs 1 and 3 information and the AO, which strongly implicates the intermediate events and therefore lessens the burden of evidence required to establish KE 2 and KE 4. Evidence for DINP PPARα activation and increased cell proliferation is incontrovertible, and thus, a change in cell signaling pathways (KE 2) is self‐evident. Likewise, evidence for DINP cell proliferation and adenoma and carcinoma formation in the absence of overt chronic cytotoxicity implies the presence of preneoplastic foci (KE 4). This comprehensive MOA evaluation advances previous assessments presented by regulatory agencies which concluded that DINP acts through the PPARα MOA for rodent hepatocarcinogenesis.

Expert panels and independent experts have largely concluded that the PPARα MOA for liver tumors lacks human relevance (Corton et al. 2014, 2018; Felter et al. 2018; Yamada et al. 2025). Therefore, several agencies have reported that DINP is likely not carcinogenic to humans. For example, Health Canada has argued that phthalates operate through the PPARα MOA for the rodent liver tumors and that the carcinogenic potential in humans is “most likely not relevant at environmentally meaningful exposure levels.” Also, ECHA reported that the PPARα MOA was of “little or unclear relevance to humans” and therefore did not consider DINP's carcinogenicity in their reviews (ECHA 2013; HC 2015). However, US EPA (2025a) has stated that preventing PPARα activation would protect against liver tumors in humans, suggesting that the rodent hepatic tumors may have human relevance. In contrast, the current independent evaluation considered the key questions in defining the human relevance of an MOA as previously defined (Boobis et al. 2006, 2008; Meek et al. 2003). This analysis demonstrated that although the WoE supports this MOA in rats and mice exposed to DINP, the KEs are not qualitatively plausible in humans, which is supported by DINP‐specific evidence that KE 2, KE 3, KE 4, and the AO do not occur in humans and nonhuman primates exposed to DINP.

Based on the data presented herein, DINP acts through the PPARα MOA to induce liver tumors in rats and mice. However, this PPARα MOA lacks human relevance broadly, and the body of DINP‐specific data, particularly in nonhuman primates, indicates a lack of human relevance. For these reasons, the liver tumors observed in rodents following exposure to DINP should not form the basis for deriving toxicity criteria, drawing human‐relevant carcinogenicity conclusions on DINP, or conducting risk evaluations in human health risk assessment.

## Funding

This work was funded by the ExxonMobil Biomedical Sciences, Inc. The contents of this manuscript solely reflect the views of the authors.

## Conflicts of Interest

The authors were employed by ToxStrategies LLC, a consulting firm that provides services on toxicology and risk assessment issues to private and public organizations, during the analysis and preparation of this manuscript. The work reported in this article was conducted during the normal course of employment. The sponsors were provided an opportunity to review the manuscript prior to submission. The purpose of this review was for the authors to receive input on the clarity of the science. The analyses, interpretation of research findings, and manuscript writing, formatting, and submission were conducted solely by the authors, and the conclusions and professional judgments were not subject to funders' control.

## Supporting information


**Table S1:** Summary of toxicological studies identified that examined the KE 1 associative event: peroxisomal β‐oxidation (PBOX) and associated enzyme activity (PCO, ACO) in rodents and nonhuman primates and human models.
**Table S2:** Summary of toxicological studies identified that examined the KE 3 in rodents and nonhuman primates and human models.

## Data Availability

Data sharing is not applicable to this article as no datasets were generated or analyzed during the current study.
