# Supplementary material for: Mode‐of‐Action and Human Relevance Assessment for Diisononyl Phthalate‐Induced Liver Tumors in Rodents
Source: J Appl Toxicol. 2026 May 5;46(12):4264–87. doi: 10.1002/jat.70223 (PMC13727332; doi:10.1002/jat.70223)
Supplement: Supplementary file 1 [file JAT-46-4264-s001.docx]

**Table S1**. Summary of toxicological studies identified that examined the KE 1 associative event: peroxisomal β-oxidation (PBOX) and associated enzyme activity (PCO, ACO) in rodents and non-human primates and human models

| **Model** | **Route, duration** | **Doses/Concentrations** | **Key Findings** | **Reference** |
| --- | --- | --- | --- | --- |
| **Rodents** | | | | |
| *In vivo* | | | | |
| Rat, F344 (M) | Diet, 1 weeks | 0, 2%  (~1700 mg/kg/day by EPA) | - ↑ relative and absolute liver weight at 1700 mg/kg/day - ↓ serum triglycerides and cholesterol | Biodynamics 1982a |
| Rat, F344 (M & F) | Diet, 3 weeks | 0, 0.6, 1.2, 2.5%  (reported by authors as ~369, 1192, 2195 mg/kg/day [M], ~607, 1193, 2289 mg/kg/day [F]) | - ↑ PCO activity at ≥1.2% (≥1192 mg/kg/day) in M and F - ↑ peroxisome proliferation at 2195 mg/kg/day in males and 2289 mg/kg/day in females - ↑ eosinophilia of cytoplasm in males at ≥1192 mg/kg/day and females at 2289 mg/kg/day - ↑ relative and absolute liver weight at ≥369/607 mg/kg/day - ↓ serum triglycerides in males at ≥369 mg/kg/day and ↑ in females at ≥1193 mg/kg/day | BIBRA 1986; Lin et al. 1987 |
| Rat, SD (M) | Gavage, 2 weeks | 0, 500 mg/kg/day | - ↔ relative liver weight - ↑ serum triglycerides but ↔ in cholesterol or HDL | Kwack et al. 2010 |
| Rat, F344 (M) | Diet, 2 and 4 weeks | 0, 1000, 12000 ppm  (~75, 900 mg/kg/day estimated by EPA) | - ↑ PBOX at 900 mg/kg/day at 2 and 4 weeks - ↑ relative liver weight at 900 mg/kg/day at 4 weeks; ↑ relative liver weight at 2 weeks for DINP-A but not DINP-1 | Smith et al. 2000 |
| Rat, SD (M) | Gavage, 4 weeks | 0, 500 mg/kg/day | - ↑ relative liver weight - ↑ serum triglycerides but ↔ in cholesterol or HDL | Kwack et al. 2009 |
| Rat, F344 (M & F) | Diet, 4 weeks | 0, 0.2, 0.67, 2.0 %  (mg/kg/day equivalents not provided) | - ↑ relative and absolute liver weight at ≥0.67% in males and females - ↓ serum triglycerides at ≥0.67% in males for DINP-I; no effects for females for DINP-I or males and females with DINP-II; ↑ cholesterol in males at 2% DINP-I, but no effects in females or for DINP-II | Midwest Research Institute 1981a |
| Rat, F344 (M & F) | Diet, 13 weeks | 0, 1000, 3000, 6000, 10000, 20000 ppm  (~77, 227, 460, 767, 1554 mg/kg/day estimated by EPA) | - ↑ peroxisome proliferation at ≥460 mg/kg/day - ↑ eosinophilic hepatocellular hypertrophy at ≥77 mg/kg/day - ↑ relative and/or absolute liver weight at ≥227 mg/kg/day - ↓ serum triglycerides at ≥460 mg/kg/day but ↔ cholesterol | Biodynamics 1982b |
| Rat, SD (M &F) | Diet, 13 weeks | 0, 0.3%, 1.0%  (~201, 690 mg/kg/day [M] and ~251, 880 mg/kg/day [F]) | - ↑ eosinophilic hepatocellular hypertrophy at ≥201 mg/kg/day^D^ - ↑ relative and absolute liver weight at ≥251 mg/kg/day in females and 690 mg/kg/day in males ^D^ - ↓ serum triglycerides at ≥201/251 mg/kg/day but ↔ cholesterol ^D^ | Biodynamics 1982c |
| Rat, albino, (M&F) | Diet, 13 weeks | 0, 50, 150, 500 mg/kg/day | - ↑ hepatocellular hypertrophy at 500 mg/kg/day - ↑ relative and/or absolute liver weight at 500 mg/kg/day | Hazleton 1971 |
| Rat, F344 (M & F) | Diet, 13 weeks | 0, 2500, 5000, 10000, 20000 ppm  (reported by authors as ~135, 276, 563, 1231 mg/kg/day [M] and 167, 347, 694, 1406 mg/kg/day [F]) | - ↑ hepatocellular enlargement at 1231/1406 mg/kg/day - ↑ relative and/or absolute liver weight in males at ≥135 mg/kg/day and females at ≥347 mg/kg/day | Hazleton 1991b |
| Rat, SD, (M & F) | Diet; 10 weeks prior to mating, throughout mating, gestation, and lactation for one generation | 0, 0.5, 1.0, 1.5%  (mg/kg/day equivalents vary by duration) | - ↑ eosinophilia of cytoplasm in parental animals - ↑ liver weight in males and females at ≥0.5% | Waterman et al. 2000 |
| Rat, SD, (M & F) | Diet; 10 weeks prior to mating, throughout mating, gestation, and lactation for two generations | 0, 0.2, 0.4, 0.8%  (mg/kg/day equivalents vary by duration) | - ↑ eosinophilia of cytoplasm in parental animals - ↑ liver weight in parental males at 0.8% and females at ≥0.4% and in second generation females at 0.8% | Waterman et al. 2000 |
| Rat, F344 (M & F) | Diet, ≤104 weeks | 0, 500, 1500, 6000, 12000 ppm  (~29, 88, 359, 733 mg/kg/day [M], ~112, 336, 910, 1888 mg/kg/day [F] estimated by EPA) | - ↑ PCO activity at 1, 2, 13, 78, 104 weeks at 733 mg/kg/day (M) and ≥910 mg/kg/day (F) [lowest dose examined] - ↑ hepatocellular enlargement and cytoplasmic eosinophilia at 2 (enlargement only), 13, 78, and 104 weeks at 733 mg/kg/day (M) and 1888 mg/kg/day (F) but not at 1 week - ↑ relative and absolute liver weight at 1, 2, 13, 78, and 104 weeks in males and females at ≥359/910 mg/kg/day | Covance 1998b |
| Rat, F344 (M & F) | Diet, ≤104 weeks | 0, 0.03, 0.3, 0.6%  (reported by authors as ~15, 152, 307 mg/kg/day [M], ~18, 184, 375 mg/kg/day [F]) | - ↔ peroxisome proliferation - ↑ in “subtle” hepatocellular enlargement at 18 and 24 months at 307/375 mg/kg/day but not at 6 or 12 months - ↑ relative and absolute liver weight at 6, 12, 18, and 24 months in males and females at ≥152/184 mg/kg/day - ↓ triglycerides and/or cholesterol in females at 375 mg/kg/day at 6 and 12 but not 18 or 24 months; ↑ cholesterol in males at 6 months but not at 12, 18, or 24 months and ↓ triglycerides at 12 but not 6, 18, or 24 months | Biodynamics 1986; Lington et al. 1997 |
| Rat, SD (M & F) | Diet, ≤104 weeks | 0, 500, 5000, 10000 ppm  (~27, 271, 553 mg/kg/day [M] and ~33, 331, 672 mg/kg/day [F]) | - ↑ relative and absolute liver weight in males at 553 mg/kg/day at 12 and 24 months and females at 672 mg/kg/day at 12 months and ≥331 mg/kg/day at 24 months - ↔ serum triglycerides or cholesterol at 6, 12, 18, or 24 months | Biodynamics 1987 |
| Mouse, B6C3F1 (M) | Diet, 2 and 4 weeks | 0, 500, 6000 ppm  (~100, 1200 mg/kg/day estimated by EPA) | - ↑ PBOX at 1200 mg/kg/day at 2 and 4 weeks - ↑ relative liver weight at 1200 mg/kg/day at 2 and 4 weeks | Smith et al. 2000 |
| Mouse, B6C3F1 (M & F) | Diet, 4 weeks | 0, 500, 1500, 4000, 8000 ppm  (reported by authors as ~100, 300, 800,1600 mg/kg/day) | - ↑ PCO activity at ≥300 mg/kg/day at 4 weeks (only time examined) - ↑ peroxisome area at ≥300 mg/kg/day in males and females at 4 weeks (only time examined); ↑ in peroxisomes of larger size categories^C^ with increasing dose - ↑ relative and absolute liver weight in males and females at ≥800 mg/kg/day at 1 and 4 week; increase in absolute liver weight in males and relative liver weight in females at ≥300 mg/kg/day at 4 weeks | Kaufmann et al. 2002 |
| Mouse, B6C3F1 (M & F) | Diet, 4 weeks | 0, 3000, 6000, 12500, 25000 ppm (reported by authors as ~601, 1308, 2678, 7560 mg/kg/day [M] and 757, 1649, 3280, 7001 mg/kg/day [F]) | - ↑ hepatocytomegaly in males at ≥601 mg/kg/day and females at ≥1649 mg/kg/day - ↑ relative and absolute liver weight at in males at ≥601 mg/kg/day and females at ≥1649 mg/kg/day | Hazleton 1991a |
| Mouse, B6C3F1 (M & F) | Diet, 4, 31, and 90 days | 0, 1500, 4000, 10000, 20000 ppm  (~0, 365, 972, 2600, 5770 mg/kg/day estimated by EPA) | - ↑ PCO activity at 365 and 2600 mg/kg/day at 4, 31, and 90 days of - ↑ hepatocellular enlargement at 31 days at 2600 mg/kg/day (only concentration tested) and 90 days at ≥2600 mg/kg/day - ↑ in relative liver weight at 90 days and ≥972 mg/kg/day | Hazleton 1992 as cited by US EPA 2025a; Hazleton 1992 ancillary report |
| Mouse, B6C3F1 | Diet, 2 weeks | 0, 150, 500, 1500, 4000, 8000 ppm  (~600, 1200 mg/kg/day at two highest concentrations estimated by EPA, other mg/kg/day not provided) | - ↑ PCO activity at ≥600 mg/kg/day - ↑ in relative liver weight at ≥600 mg/kg/day in males ≥1500 ppm in females | Valles et al. 2003 |
| Mouse, B6C3F1, SV129, and PPARα-null | Diet, 1 and 3 weeks | 0, 8000 ppm  (~1200 mg/kg/day estimated by EPA) | - ↑ PCO activity at 1200 mg/kg/day in wt strains at both 1 and 3 weeks, no induction in PPARα-null mice - ↑ protein expression of ACO and other PBOX associated enzymes in SV129 mice at 1200 mg/kg/day at both 1 and 3 weeks, which was not observed in PPARα-null mice - ↑ in relative liver weight in wt mice at 1 and 3 weeks but not PPARα-null mice except females at 3 weeks | Valles et al. 2003 |
| Mouse, SV129 and PPARα-null (M) | Gavage, 1 week | 0, 1000 mg/kg/day | - ↑ Protein expression of ACO and other PBOX associated enzymes in SV129 mice at 1000 mg/kg/day, which was not observed in PPARα-null mice - ↔ relative liver weight | Valles et al. 2003 |
| Mouse, BALB/c (M) | Oral, 4 weeks | 0, 20, 200 mg/kg/day | - ↑ triglycerides, LDL, and HDL at 200 mg/kg/day and cholesterol at 20 mg/kg/day | Ore et al. 2022 |
| Mouse, B6C3F1 (M) | Dermal, 4 weeks | 0, 0.02, 0.2, 2, 20, 200 mg/kg/day | - ↑ relative liver weight at 200 mg/kg/day | Liang et al. 2020 |
| Mouse, B6C3F1 (M & F) | Diet, ≤104 weeks | 0, 500, 1500, 4000, 8000 ppm  (~90, 276, 742, 1560 mg/kg/day [M] and ~112, 336, 910, 1888 mg/kg/day [F]) | - ↑ PCO activity at in males and females 1560/1888 mg/kg/day (only dose tested) for 78 and 104 weeks - ↑ hepatocellular enlargement and cytoplasmic eosinophilia at 1560/1888 mg/kg/day at 79 and 104 weeks - ↑ relative and/or absolute liver weight in males at ≥742 mg/kg/day and females at 1888 mg/kg/day at 78 weeks; increase in relative and absolute liver weight in males at ≥742 mg/kg/day in males and 1888 mg/kg/day in females at 104 weeks | Covance 1998a |
| *In vitro* | | | | |
| mPPARα in 3T3-LI cells | 24 hours | 0, 3, 10, 30, 100, 200 uM (MINP) | - ↑ mPPARα activation at ≥3 uM | Bility et al. 2004 |
| Primary rat hepatocytes F344 (M) | 24 or 48 hours | 0, 250, 1000 uM (DINP) | - Transcriptomic profile of DINP was consistent with that of the prototypical PPARα agonists WY14643 and clofibrate | De Abrew et al. 2015 |
| Primary rat hepatocytes, F344 (M) | Duration NR | 0, 250, 500, 750 uM (DINP) | - ↑ PBOX at ≥250 uMA | Hasmall et al. 1999 |
| Primary rat hepatocytes, F344, (M) | 24 and 72 hours | 0, 150, 200, 250 uM (MINP) | - ↑ PCO activity at ≥150 uM | Shaw et al. 2002 |
| Primary rat hepatocytes, F344 (M) | 72 hours | 0, 50, 100, 200, 300, 500 uM (MINP) | - ↑ PBOX at ≥200 uMB | Kamendulis et al. 2002 |
| Primary rat hepatocytes, Wistar (M) | 72 hours | 0, 0.1, 0.25, 0.5 mM (DINP) | - ↔ PCO activity attributed to poor metabolic capacity of the culture | Benford et al. 1986 |
| Primary rat hepatocytes, Wistar (M) | 72 hours | 0, 0.1, 0.25, 0.5 mM (MINP) | - ↑ PCO activity at ≥0.1 mM | Benford et al. 1986 |
| Primary mouse hepatocytes, B6C3F1 (F) | 72 hours | 0, 10, 50, 100, 200, 300 uM (MINP) | - ↑ PBOX at ≥50 uM | Kamendulis et al. 2002 |
| **Humans and non-human primates** | | | | |
| *In vivo* | | | | |
| Monkey, cynomolgus (M) | Gavage, 14 days | 0, 500 mg/kg/day | - ↔ PBOX - ↔ liver histology indicative of hepatocyte hypertrophy - ↔ liver weight - ↔ serum triglycerides | Pugh et al. 2000 |
| Monkey, cynomolgus (M & F) | Gavage, 13 weeks | 0, 100, 500, 2500 mg/kg/day | - ↔ PCO activity - ↔ liver histology indicative of hepatocyte hypertrophy - ↔ liver weight | Hall et al. 1999/ Huntingdon 1998 |
| *In vitro* | | | | |
| hPPARα in 3T3-LI cells | 24 hours | 0, 3, 10, 30, 100, 200 uM (MINP) | - ↑ hPPARα activation at ≥10 uM | Bility et al. 2004 |
| hPPARα in COS-1 cells | 24 hours | 0, 0.1, 1, 10, 100 uM (MINP) | - ↑ hPPARα activation at ≥100 uM | Laurenzana et al. 2016 |
| hPPARα in COS-1 cells | 24 hours | 0, 1 uM (DINP) | - ↔ hPPARα activation | Laurenzana et al. 2016 |
| Primary human hepatocytes (NR) | Duration NR | 0, 250, 500, 750 uM (DINP) | - ↔ PBOX | Hasmall et al. 1999 |
| Primary human hepatocytes (M) | 24 and 72 hours | 0, 150, 200, 250 uM (MINP) | - ↔ PCO activity | Shaw et al. 2002 |
| HLEC-04 cell line (M) | 72 hours | 0, 10, 50, 100, 200, 300 uM (MINP) | - ↔ PBOX | Kamendulis et al. 2002 |
| Primary monkey hepatocytes (M) | 72 hours | 0, 10, 50, 100 uM (MINP) | - ↔ PBOX | Kamendulis et al. 2002 |
| Primary monkey hepatocytes (M) | 72 hours | 0, 0.1, 0.25, 0.5 mM (DINP) | - ↔ PCO activity | Benford et al. 1986 |
| Primary monkey hepatocytes (M) | 72 hours | 0, 0.1, 0.25, 0.5 mM (MINP) | - ↔ PCO activity | Benford et al. 1986 |

Key: M: male; F: female; DINP: di-isononyl phthalate; MINP: mono-isononyl phthalate; PBOX: peroxisomal beta-oxidation; PCO: palmitoyl-CoA;
ACO: acetyl-CoA; LDL: low-density lipoprotein; HDL: high-density lipoprotein; wt: wild-type; US EPA: United States Environmental Protection Agency

^A^ Not observed at 750 uM, but cytotoxicity was not evaluated

^B^ Observed at highest concentration of MINP-2 (highest concentration tested) and 300 uM MINP-1 and MINP-3 (highest concentration tested)

^C^ Peroxisomes were categorized as size 1, 2, 3, 4, or 5, with 1 being the sizes and 5 being the largest

^D^ Water uncoupled for some rats during the 12^th^ week of exposure

**Table S2**. Summary of toxicological studies identified that examined the KE 3 in rodents and non-human primates and human models

| **Model** | **Route, duration** | **Doses/Concentrations** | **Key Findings** | **Reference** |
| --- | --- | --- | --- | --- |
| **Rodents** | | | | |
| *In vivo* | | | | |
| Rat, F344 (M) | Diet, 2 and 4 weeks | 0, 1000, 12000 ppm  (~75, 900 mg/kg/day estimated by EPA) | - ↑ replicative DNA synthesis (via BrdU) at 900 mg/kg/day at 2 weeks for DINP-1 and DINP-A, but only DINP-A at week 4 | Smith et al. 2000 |
| Rat, F344 (M & F) | Diet, ≤104 weeks | 0, 500, 1500, 6000, 12000 ppm  (~29, 88, 359, 733 mg/kg/day [M], ~112, 336, 910, 1888 mg/kg/day [F] estimated by EPA) | - ↑ mitotic activity and hepatocyte proliferation at 1 week at 733/1888 mg/kg/day; not observed at ≥2 weeks | Covance 1998b |
| Mouse, B6C3F1 (M & F) | Diet, 1, 4 weeks | 0, 500, 1500, 4000, 8000 ppm  (reported by authors as ~100, 300, 800,1600 mg/kg/day) | - ↑ hepatocellular proliferation after 1 and 4 weeks at ≥100 mg/kg/day in males; ↑ hepatocellular proliferation at 1 week at ≥800 mg/kg/day in females but no change at 4 weeks - ↔ in apoptosis at 4 weeks and non-dose dependent changes at 1 week | Kaufmann et al. 2002 |
| Mouse, B6C3F1 (M & F) | Diet, 4, 31, and 90 days | 0, 1500, 4000, 10000, 20000 ppm  (~0, 365, 972, 2600, 5770 mg/kg/day estimated by EPA) | - ↔ replicative DNA synthesis at 4, 31, or 90 days | Hazleton 1992 as cited by US EPA 2025a; Hazleton 1992 ancillary report |
| Mouse, B6C3F1, SV129, and PPARα-null (F) | Diet, 1 weeks | 0, 8000 ppm  (~1200 mg/kg/day estimated by EPA) | - ↑ replicative DNA synthesis (via BrdU) at 1 week in wt strains, but no proliferation in PPARα-null mice | Valles et al. 2002 |
| Mouse, B6C3F1 (M) | Diet, 2 and 4 weeks | 0, 500, 6000 ppm  (~100, 1200 mg/kg/day estimated by EPA) | - ↑ hepatocellular proliferation (via BrdU) at 1200 mg/kg/day DINP-1 but not DINP-A at 2 weeks; ↔ at week 4 | Smith et al. 2000 |
| Mouse, B6C3F1 (M & F) | Diet, ≤104 weeks | 0, 500, 1500, 4000, 8000 ppm  (~90, 276, 742, 1560 mg/kg/day [M] and ~112, 336, 910, 1888 mg/kg/day [F]) | - No biologically relevant change in DNA content at 79 or 104 weeks in males and females - ↔ hepatocellular proliferation at 79 or 105/106 weeks | Covance 1998a |
| *In vitro* | | | | |
| Primary rat hepatocytes, F344 (M) | Duration NR | 0, 250, 500, 750 uM (DINP) | - ↑ hepatocyte proliferation at ≥250 uM - ↓ apoptosis at ≥250 uM | Hasmall et al. 1999 |
| Primary rat hepatocytes, F344, (M) | 24 and 72 hours | 0, 150, 200, 250 uM (MINP)  0, 150, 175, 200, 225, 250 uM (MINP) – proliferation only | - ↑ hepatocyte proliferation at ≥150 uM - ↓ apoptosis at ≥150 uM | Shaw et al. 2002 |
| **Humans and non-human primates** | | | | |
| *In vivo* | | | | |
| Monkey, cynomolgus (M) | Gavage, 14 days | 0, 500 mg/kg/day | - ↔ hepatocyte proliferation | Pugh et al. 2000 |
| *In vitro* | | | | |
| Primary human hepatocytes | Duration NR | 0, 250, 500, 750 uM (DINP) | - ↔ hepatocyte proliferation - ↔ apoptosis | Hasmall et al. 1999 |
| Primary human hepatocytes (M) | 24 and 72 hours | 0, 150, 200, 250 uM (MINP)  0, 150, 175, 200, 225, 250 uM (MINP) – proliferation only | - ↔ hepatocyte proliferation - ↔ apoptosis | Shaw et al. 2002 |

Key: M: male; F: female; DINP: di-isononyl phthalate; MINP: mono-isononyl phthalate; wt: wild-type; US EPA: United States Environmental Protection Agency;
BrdU: 5-bromo-2’-deoxyuridine
